# CLDN5 as a novel modulator of podocyte adhesion to extracellular matrix *via* β1-integrin binding

**DOI:** 10.1016/j.jbc.2026.111163

**Published:** 2026-01-13

**Authors:** Chao Wang, Jingyi Han, Baozhen Fan, Yaxi Shen, Yanan An, Feng Kong, Nan Ge, Xiulin Zhang, Hao Liu, Mingxia Wang, Hui Sun, Chengjun Zhou, Shengtian Zhao, Yongfeng Gong

**Affiliations:** 1 Department of Urology, The Second Hospital, Cheeloo College of Medicine, Shandong University, Jinan, Shandong, China; 2 Department of Urology, Qilu Hospital, Cheeloo College of Medicine, Shandong University, Jinan, Shandong, China; 3 Shandong Engineering Research Center of Molecular Medicine for Renal Diseases, Yantai, Shandong, China; 4 Laboratory of Tight Junction, Binzhou Medical University, Yantai, Shandong, China; 5 Department of Thoracic Surgery, Qilu Hospital of Shandong University, Jinan, Shandong, China; 6 Department of Urology, Yantai Affiliated Hospital of Binzhou Medical University, Yantai, Shandong, China; 7 Department of Urology, Binzhou Medical University Hospital, Binzhou, Shandong, China; 8 Department of Physiology, Binzhou Medical University, Yantai, Shandong, China; 9 Shandong Provincial Engineering Laboratory of Urologic Tissue Reconstruction, Jinan, Shandong, China; 10 Department of Central Laboratory, Shandong Provincial Hospital Affiliated to Shandong First Medical University, Jinan, Shandong, China; 11 Department of Pathology, The Second Hospital, Cheeloo College of Medicine, Shandong University, Jinan, Shandong, China

**Keywords:** podocytes, CLDN5, β1-integrin, HUWE1

## Abstract

The intricate glomerular filtration barrier relies on robust podocyte adhesion to the glomerular basement membrane (GBM), a process critical for kidney function and often compromised in chronic kidney diseases. Here, we reveal that the four-transmembrane protein CLDN5 is an important molecule regulating podocyte adhesion. Utilizing super-resolution imaging, we pinpoint CLDN5’s localization at the podocyte-GBM interface, where it notably colocalizes with β1-integrin. CLDN5 deletion in podocytes profoundly impairs cell adhesion, spreading, and resistance to mechanical stress *in vitro*. Mechanistically, CLDN5 forms a stable complex with β1-integrin, and its loss leads to a significant reduction in β1-integrin protein levels coupled with aberrant localization. CLDN5 binds to the intracellular domain of β1-integrin *via* its intracellular loop and C-terminal domains, thereby impeding HUWE1-mediated ubiquitination at lysine K774 and subsequent proteasomal degradation as well as ensuring proper membrane localization of β1-integrin. The protective role of CLDN5 in maintaining podocyte integrity is supported by *in vivo* studies, demonstrating markedly exacerbated renal injury in *Cldn5*-KO mice subjected to hypertensive and adriamycin-induced injury models. These findings not only broaden our understanding of the extra-junctional roles of claudin proteins but also provide critical molecular insights into the complex mechanisms by which podocytes maintain integrity and withstand mechanical forces within the glomerulus.

The unique position of podocyte within the glomerulus subjects it to continuous biomechanical stress, necessitating an exceptionally strong and dynamic adhesion to the glomerular basement membrane (GBM) to preserve the integrity of the glomerular filtration barrier and ensure effective blood ultrafiltration (1). Podocyte adhesions to the GBM are predominantly facilitated by the extracellular interaction of integrin heterodimers composed of individual α and β subunits (2). Podocytes express various integrin receptors, with the laminin-binding α3β1-integrin serving as the primary mediator of their adhesion to the GBM (3). These heterodimers serve to connect GBM components with the intracellular cytoskeleton, thereby governing various aspects of podocyte function such as adhesion, migration, differentiation, and survival. Crucially, the functional efficacy and regulatory capacity of integrin-mediated adhesion are significantly enhanced by their intimate association and precise communication with other key transmembrane and cytoplasmic proteins, which collectively form sophisticated focal adhesion complexes. While several transmembrane proteins are known to enhance integrin-mediated cell adhesion, the specific contributions and precise mechanisms of many other such transmembrane proteins under physiological conditions and in the pathogenesis of glomerular diseases are not yet fully understood.

In podocytes, as in other cell types, the tetraspanin CD151 associates with adhesion receptors from the integrin family and regulates integrin-mediated cell adhesion to the extracellular matrix (ECM) (4, 5, 6, 7, 8, 9). CD151 increases the strength of podocyte adhesion to the GBM *via* α3-integrin engagement with laminin-α5β2γ1 at the base of foot processes (6). Global deletion of *Cd151* in mouse models on the FVB background results in early and severe proteinuria, accompanied by focal segmental glomerulosclerosis and eventual kidney failure (7). *Cd151*-knockout mice on the C57BL/6 background do not spontaneously develop kidney phenotypes but exhibit nephrotic range proteinuria alongside hypertension (8). Claudins (CLDNs), as distant relatives of the four-transmembrane protein family, constitute a family of 27 transmembrane proteins that serve as critical components within the tight junction complex, facilitating both homo- and heterotypic interactions between neighboring cells to establish specialized paracellular barriers in epithelia and endothelia (10). A notable feature of CLDNs is their diverse localization—not only at the apical tight junction complex, but also on basolateral membranes, within intracellular vesicles, or in the nucleus—indicating their involvement in a wide range of traditional and emerging non-traditional functions in health and disease (11, 12, 13, 14, 15, 16).

Mature podocytes, which are highly specialized epithelial cells, lack tight junctions and are connected by intercellular junctions known as slit diaphragms. Podocytes are atypical epithelial cells that evolve from primordial columnar epithelial cells connected by an apical junction complex containing both tight junctions and adhesion junctions (17). As these epithelial cells develop into podocytes with an intricate network of foot processes, the early apical tight junctions and adhesion junctions migrate basally and ultimately morph into a single slit diaphragm that connects the interdigitating foot processes of adjacent podocytes (18). Although the tight junction structure disappeared in mature podocytes, the expression of tight junction proteins is maintained near the mature slit diaphragm (19, 20, 21). Early cosedimentation analysis of glomerular lysates *via* sucrose gradient centrifugation revealed that the tight junction proteins JAM-A, occludin, cingulin, ZO-1, and CASK cofractionate with slit diaphragm markers nephrin, podocin, CD2AP, and Neph1 in the normal glomerulus (19). In contrast, CLDN5 cofractionates with the apical membrane protein podocalyxin, the basal protein β1-integrin, and the slit diaphragm proteins (19), implying its apical and basal expression. An antibody-based immunoelectron microscopy study indicated that CLDN5 is not only localized within the cell body but also exhibits a prominent distribution along the basolateral membrane in podocytes from wild-type mouse kidneys (22). These findings suggest that CLDN5 is also localized to sites outside the intercellular junction complex, unlike other tight junction proteins typically expressed within the slit diaphragm complex in podocytes, highlighting its diverse and complex roles in podocyte biology.

We have previously demonstrated that the absence of CLDN5 exacerbates podocyte disease by transcriptionally downregulating WNT inhibitor factor-1 (WIF1) and activating WNT signaling (23). However, while genetic deletion of *Wif1* in podocytes resulted in a comparable glomerular phenotype, it was notably less severe than in mice with podocyte-specific *Cldn5* deletion, suggesting that CLDN5 likely exerts additional protective functions in podocytes beyond WIF1-mediated WNT signaling regulation (23). This study reveals that CLDN5 plays an extra-junctional role in regulating podocyte adhesion by directly interacting with and stabilizing β1-integrin at the podocyte-GBM interface, which protects β1-integrin from HUWE1-mediated ubiquitination and promotes its membrane localization. This interaction enhances podocyte adhesion and resistance to mechanical stress, thereby preventing glomerular injury in models of hypertensive and adriamycin-induced nephropathy. Thus, our study demonstrates that CLDN5 may work in conjunction with integrins to strengthen podocyte adhesion to the GBM, counteracting the mechanical forces generated by urine flow and continuous stretching. This broadens our understanding of the localization and function of extra-tight junction claudin proteins and provides new molecular insights into the mechanisms that enable podocytes to withstand mechanical stress within the glomerulus.

## Results

### CLDN5 localizes at the podocyte–GBM interface and enhances podocyte adhesion

**Figure 1:**
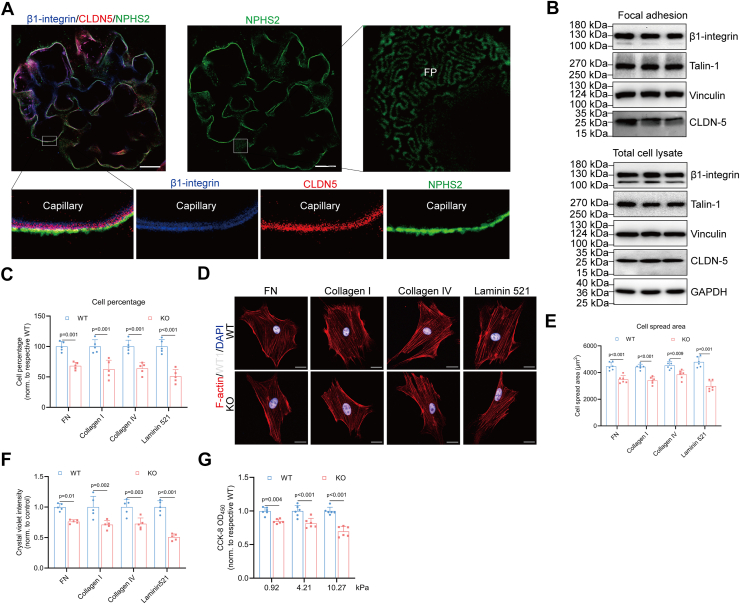
**CLDN5 localizes at the podocyte-GBM interface and enhances podocyte adhesion**. *A*, high-resolution expansion microscopy of intraglomerular localization of β1-integrin (*blue*), CLDN5 (*red*), and podocin (NPHS2, *green*). Scale bars, 20 μm. *B*, Western blot analysis of CLDN5 in the focal adhesion complexes and whole cell lysate with β1-integrin, talin-1, and vinculin serving as markers to validate the successful fractionation. *C–E*, Cell percentage (*C*, n = 5) and spread area (*D* and *E*, n = 6 biologically independent animals, 10 cells per animal) of WT and *Cldn5*-KO podocytes on specific ECM ligands. Scale bars, 20 μm. *F*, relative number of adherent podocytes on specific ECM ligands measured by crystal violet staining (n = 5). *G*, podocyte adhesion to matrices with varying mechanical properties assessed using the CCK-8 assay (n = 6). Data are presented as mean ± SD. Statistical significance was determined by two-way ANOVA followed by Tukey’s post-test. FP, foot process.

Previous studies using cosedimentation analysis of glomerular lysates *via* sucrose gradient centrifugation, as well as immunogold transmission electron microscopy, have reported both apical and basal localization of CLDN5 in podocytes (19, 22). To test whether CLDN5 is localized within adhesion structures, we examined the distribution of CLDN5 *via* expansion microscopy and double-labeling immunofluorescent experiments which can create effective super resolution imaging. Our results revealed two distinct layers of β1-integrin within the GBM, consistent with its expression in both podocytes and endothelial cells (Fig. 1*A*). Notably, we observed clear colocalization of CLDN5 and β1-integrin on the podocyte surfaces along the GBM (Fig. 1*A*), where the integrin-associated focal adhesion complex anchors podocytes to the ECM at the basal side. Western blot analysis of focal adhesion-enriched cell fractions and total cell lysates further confirmed that CLDN5 is enriched in focal adhesions of podocytes (Fig. 1*B*).

Next, we investigated whether CLDN5 is involved in the regulation of podocyte adhesion using the podocyte-specific conditional *Cldn5* knockout mice (*Cldn5*-KO). To examine the effect of CLDN5 deletion on podocyte adhesion and spreading on various ECM ligands, primary podocytes isolated from control and *Cldn5*-KO mice were seeded on plates coated with fibronectin, type I collagen, type IV collagen, or laminin-α5β2γ1. After enzymatic dissociation, a single-cell suspension of podocytes replated on ECM began to attach and spread. We noted a marked decrease in both the percentage and the area of cell spreading of isolated mutant podocytes compared to the control group (Fig. 1, *C* and *D*), with no significant change in cell volume (Fig. S1, *A* and *B*). Furthermore, there was a significant difference in the number of cells adhering to these matrices after 2 h between CLDN5-positive and -negative podocytes (Fig. 1*E*). Given that podocytes *in vivo* are fully spread and must endure transcapillary filtration pressure, we proceeded to culture podocytes on gelatin methacrylate-based hydrogels (GelMA) with different stiffness (0.92 kPa, 4.21 kPa, and 10.27 kPa) and quantified cellular attachment by CCK-8 assay. CLDN5-deficient podocytes exhibited significantly reduced tolerance to extracellular mechanical stress compared to wild-type podocytes (Fig. 1*F*). Hence, *Cldn5* deletion significantly impaired podocyte attachment and spreading on a variety of matrices and substrates with different rigidities.

### Loss of podocyte CLDN5 dysregulates β1-integrin expression and localization

**Figure 2:**
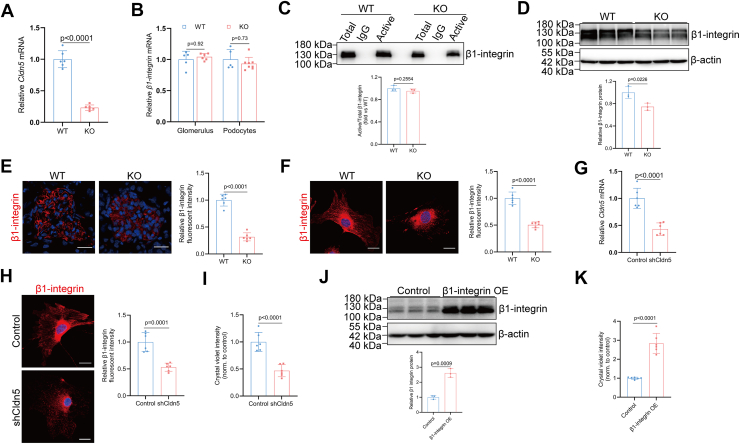
**Loss of podocyte CLDN5 dysregulates β1-integrin expression and localization**. *A*, qRT-PCR analysis of CLDN5 mRNA in isolated podocytes from WT and *Cldn5*-KO mice at 4 weeks of age (n = 6). *B*, qRT-PCR analysis of β1-integrin mRNA abundance in isolated glomerulus or podocyte from WT and *Cldn5*-KO mice at 4 weeks of age (n = 5). *C*, representative Western blot of β1-integrin in total cell lysates (‘Total’) and immunoprecipitated fractions (‘Active’), and densitometric analysis representing the ratio of Active to Total β1-integrin (n = 3). *D*, Western blot with densitometric analysis of β1-integrin in isolated glomerulus from WT and *Cldn5*-KO mice at 4 weeks of age (n = 3). *E* and *F*, immunofluorescence and quantitative analysis of β1-integrin in kidney sections (*E*), or in the isolated podocytes (*F*) from WT and *Cldn5*-KO mice at 4 weeks of age (n = 6). Scale bars, 20 μm. *G*, qRT-PCR analysis of CLDN5 mRNA in isolated WT podocytes treated with lentivirus carrying either control- or *Cldn5*-shRNA (n = 6). *H*, immunofluorescence and quantitative analysis of β1-integrin in isolated WT podocytes treated with lentivirus carrying either control- or *Cldn5*-shRNA (n = 6). Scale bars: 20 μm. *I*, the intensity of crystal *violet* staining, used here to quantify the relative number of adherent cells (n = 6), indicated reduced cell adhesion in WT primary podocytes transduced with *Cldn5*-shRNA lentivirus compared to control shRNA. *J*, Western blot with densitometric analysis of β1-integrin in isolated *Cldn5*-KO podocytes treated with lentivirus carrying either control or β1-integrin coding sequence (n = 3). *K*, relative numbers of adherent cells determined by crystal *violet* staining in isolated *Cldn5*-KO podocytes treated with lentivirus carrying either control or β1-integrin coding sequence (n = 6). Data are presented as mean ± SD. Statistical significance was determined by two-tailed Student’s unpaired *t* test analysis (*A*, *C*–*K*), two-way ANOVA followed by Tukey’s post-test (*B*).

To gain insight into the mechanism by which CLDN5 regulates podocyte–ECM adhesion, we analyzed the effects of CLDN5 deficiency on expression and activation of integrins. In podocytes, integrins α3β1 bind to laminin, α1β1 and α2β1 bind to collagen, and α5β1 and α2β1 bind to fibronectin (FN) in the GBM matrix. *Cldn5*-KO podocytes exhibited reduced cell attachment and spreading on various matrices, including FN, type I collagen, type IV collagen, or laminin-α5β2γ1 (Fig. 1, *C*–*E*). Additionally, CLDN5 was found to co-localize with β1-integrin at the basolateral membrane of podocytes (Fig. 1*A*). These results collectively suggest a potential regulatory role of CLDN5 in cell adhesion *via* β1-integrin, and we next study the contribution of β1-integrin to CLDN5-mediated signaling. No significant differences were observed in the mRNA expression levels of β1-integrin from enriched glomerulus and primary podocytes harvested from control and *Cldn5*-KO mice (Fig. 2, *A* and *B*). Using antibody 9EG7 recognizing the active conformation of β1-integrin, we found a similar proportion of active β1-integrin in *Cldn5*-deficient podocytes when compared with wild-type cells (Fig. 2*C*), suggesting that a proper level of CLDN5 is not required for optimal integrin activation in podocytes. However, β1-integrin expression and localization were visibly impaired at the podocyte-GBM interface in *Cldn5*-KO mice of 4-weeks old (Fig. 2, *D*–*F*), which lacked albuminuria and glomerulosclerosis (23). Further, in contrast to control podocytes showing β1-integrin expression distinctly at the plasma membrane, *Cldn5* knockout led to a punctate expression pattern of β1-integrin with less membrane accumulation following immunostaining (Fig. 2*F*). To directly examine the role of CLDN5 in podocytes, we knocked down its expression in primary podocytes and observed a reduction in β1-integrin levels along with impaired adhesion to the laminin-α5β2γ1 matrix (Fig. 2, *G*–*I*), underscoring CLDN5’s essential role in maintaining β1-integrin-mediated adhesion. This defect was partially rescued by ectopic expression of β1-integrin, evidenced by a significant increase in cellular adhesion to the laminin-α5β2γ1 matrix (Fig. 2, *J* and *K*), suggesting that the phenotype in *Cldn5*-deficient podocytes may be attributable to decreased β1-integrin protein levels. Collectively, these findings demonstrate that *Cldn5* knockout results in reduced expression and mislocalization of β1-integrin, indicating that CLDN5 may influence podocyte attachment through β1-integrin during cell-matrix interactions.

### CLDN5 protects β1-integrin from HUWE1-mediated ubiquitination and degradation

**Figure 3:**
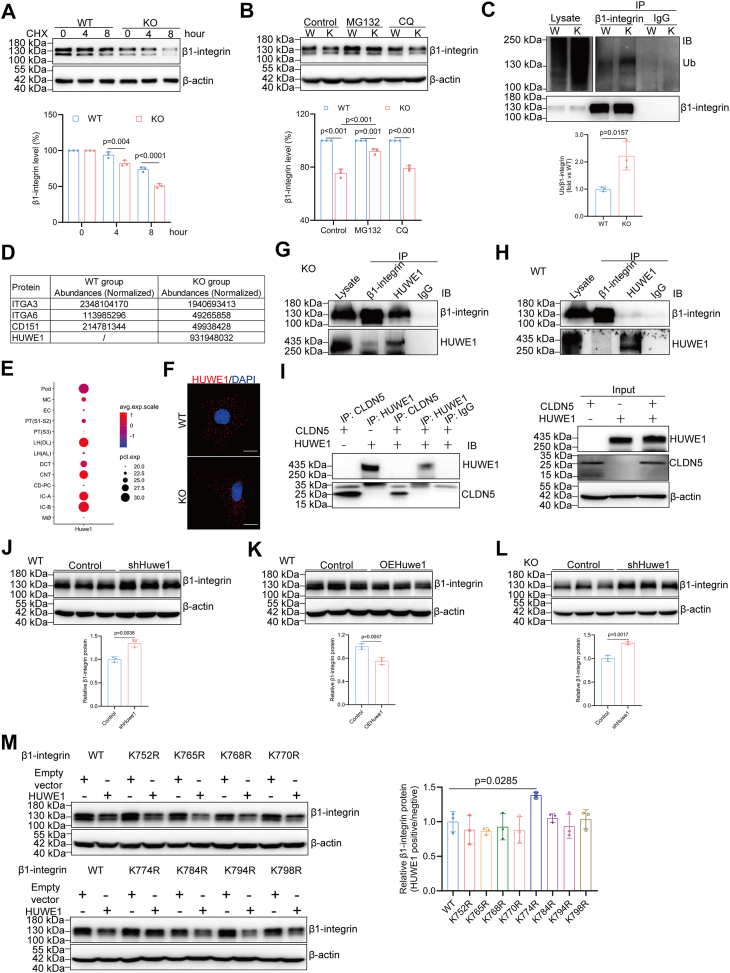
**CLDN5 protects β1-integrin from HUWE1-mediated ubiquitination and degradation**. *A*, Western blot with densitometric analysis of β1-integrin in isolated podocytes treated with 20 μg/ml cycloheximide (CHX) for 0, 4, and 8 h, CHX was used to inhibit new protein synthesis and assess β1-integrin protein stability. The line graph shows relative β1-integrin protein levels, quantified as the β1-integrin/β-actin ratio, with the 0-h point arbitrarily set to 100%. *B*, Western blot with densitometric analysis of β1-integrin in isolated podocytes treated with vehicle (control), 10 μM MG132, or 20 μM Chloroquine (CQ) for 8 h (n = 3). *C*, Ubiquitination levels of β1-integrin in isolated podocytes from WT and *Cldn5*-KO mice (n = 3). *D*, mass spectrometry analysis of β1-integrin immunocomplexes precipitated from isolated glomerulus. *E*, single-cell RNA sequencing data for *Huwe1* from healthy mouse kidneys, obtained from the Humphreys Lab's Online Analyzer for Kidney Single Cell Datasets. Pod, podocyte; MC, mesangial cell; EC, endothelial cell; PT, proximal tubule; LH (DL), descending loop of Henle; LH (AL), ascending loop of Henle; DCT, distal convoluted tubule; CNT, connecting tubule; CD-PC, collecting duct principal cell; IC-A, intercalated cells-α; IC-B, intercalated cells-β; Mø, macrophage. *F*, immunofluorescence analysis of HUWE1 in primary podocyte. Scale bars, 20 μm. (*G* and *H*) Co-immunoprecipitation (Co-IP) of CLDN5 and β1-integrin in isolated glomerulus from *Cldn5*-KO (*G*) and WT (*H*) mice to assess the interaction of endogenous CLDN5 with β1-integrin. *I*, Co-IP of CLDN5 and HUWE1 in doubly transfected HEK293 cells shows no interaction between CLDN5 and HUWE1. (*J* and *K*) Western blot with densitometric analysis of β1-integrin in the isolated WT podocytes treated with lentivirus carrying either control- or *Huwe1*-shRNA (*J*), or lentivirus carrying either control or *Huwe1* coding sequence (*K*) (n = 3). *L*, Western blot with densitometric analysis of β1-integrin in the isolated *Cldn5*-KO podocytes treated with lentivirus carrying either control- or *Huwe1*-shRNA (n = 3). *M*, cellular abundance of β1-integrin in isolated podocytes co-transfected with *Huwe1* and either WT or lysine-mutant β1-integrin gene (n = 3). For all immunoprecipitation or Co-IP analyses, antibodies used for immunoprecipitation are shown above the lanes; antibody for blot visualization is shown on the *right*. Control IPs with singly transfected cells (β1-integrin or CLDN alone) confirm specificity and exclude nonspecific binding. Whole-cell lysates (input) from singly and doubly transfected cells demonstrate expression levels of CLDNs and β1-integrin. Data are presented as mean ± SD. Statistical significance was determined by two-way ANOVA followed by Tukey’s post-test (*A*, *B*), two-tailed Student’s unpaired *t* test analysis (*C*, *J*–*L*), one-way ANOVA followed by Tukey’s post-test (*M*).

Next, we investigated the mechanism underlying the dysregulation of β1-integrin expression in *Cldn5*-null podocytes. A reduction in total β1-integrin levels was observed in both glomerular and podocyte, with no change in β1-integrin mRNA levels (Fig. 2, *B*–*F*), indicating that the decrease in β1-integrin protein levels following CLDN5 ablation is due to altered protein turnover in *Cldn5*-null podocytes. As shown in Figure 3*A*, β1-integrin protein degradation was accelerated in *Cldn5*-KO podocytes compared to wild-type podocytes, as observed 8 h after cycloheximide (CHX) treatment (Fig. 3*A*). The proteasome inhibitor MG132, rather than the lysosome inhibitor chloroquine (CQ), significantly reversed the degradation of β1-integrin (Fig. 3*B*), indicating that the proteasome plays a role in regulating β1-integrin levels in these cells. Some reports have demonstrated that the ubiquitin-proteasome system is involved in the degradation of β1-integrin (24). We hypothesised that CLDN5 may regulate β1-integrin degradation through the ubiquitin-proteasome pathway. Immunoprecipitation with an anti-β1-integrin antibody followed by Western blot analysis with an anti-ubiquitin antibody revealed elevated levels of ubiquitinated β1-integrin in *Cldn5*-KO podocytes compared to wild-type podocytes (Fig. 3*C*). Thus, the impaired β1-integrin expression caused by loss of CLDN5 is likely due to the increased ubiquitination and elevated degradation.

To identify an E3 ligase capable of regulating β1-integrin in *Cldn5*-null podocytes, we performed immunoprecipitation of β1-integrin followed by mass spectrometry analysis. In addition to the known β1-integrin binding partners, such as α3-integrin, α6-integrin, and CD151, the E3 ubiquitin ligase HECT, UBA, and WWE domain-containing E3 ubiquitin-protein ligase 1 (HUWE1) was identified in the protein complex immunoprecipitated by a β1-integrin antibody in *Cldn5*-KO podocytes (Fig. 3*D*). Single-cell RNA sequencing data from the mouse kidney revealed abundant expression of HUWE1 in podocytes (Fig. 3*E*) (25), which was further confirmed by immunofluorescence performed on primary podocytes (Fig. 3*F*). The interaction between β1-integrin and HUWE1 was validated through Western blot analysis following co-immunoprecipitation (Co-IP) experiments in CLDN5-deficient podocytes (Fig. 3*G*), strongly suggesting that β1-integrin is a target of HUWE1, potentially promoting its ubiquitination and degradation. However, we detected little to no interaction in wild-type podocytes (Fig. 3*H*), suggesting that the presence of CLDN5 inhibits the interaction between β1-integrin and HUWE1. Moreover, Co-IP experiments of CLDN5 and HUWE1 in doubly transfected HEK293 cells showed that CLDN5 had no interaction with HUWE1 (Fig. 3*I*), suggests that CLDN5 does not directly impede HUWE1 activity by binding to HUWE1 itself, instead, its inhibitory effect is likely mediated through its interaction with β1-integrin. We next examined β1-integrin protein expression levels in cultured mouse primary podocytes with modified HUWE1 expression. As shown in Figure 3*J*, knockdown of HUWE1 using shRNA significantly increased β1-integrin protein levels compared to cells transfected with negative control (NC) shRNA (Figs. 3*J* and S2*A*). Conversely, overexpression of HUWE1 markedly reduced β1-integrin protein abundance in podocytes (Figs. 3*K* and S2*B*). Further, transfection of HUWE1 shRNA recovered the level of β1-integrin compared with that of the control shRNA transfected *Cldn5*-KO podocytes (Figs. 3*L* and S2*C*). These complementary approaches demonstrate that HUWE1 negatively regulates β1-integrin expression in podocytes.

To precisely identify the HUWE1-mediated ubiquitination site on β1-integrin, we generated a panel of β1-integrin missense constructs, each bearing a substitution of arginine (R) for one of the eight lysine (K) residues located within its intracellular C-terminal domain. As depicted in Figure 3*M*, the K774R mutant uniquely demonstrated resistance to HUWE1-induced down-regulation of β1-integrin (Fig. 3*M*). This finding strongly suggests that K774 is the critical ubiquitination locus. Integrating these observations, our data confirm that depletion of CLDN5 enhances β1-integrin degradation, a process driven by the direct interaction of HUWE1 with β1-integrin, which facilitates proteasomal degradation predominantly through ubiquitination at the K774 residue.

### CLDN5 forms a complex with β1-integrin to stabilize its cell surface expression

**Figure 4:**
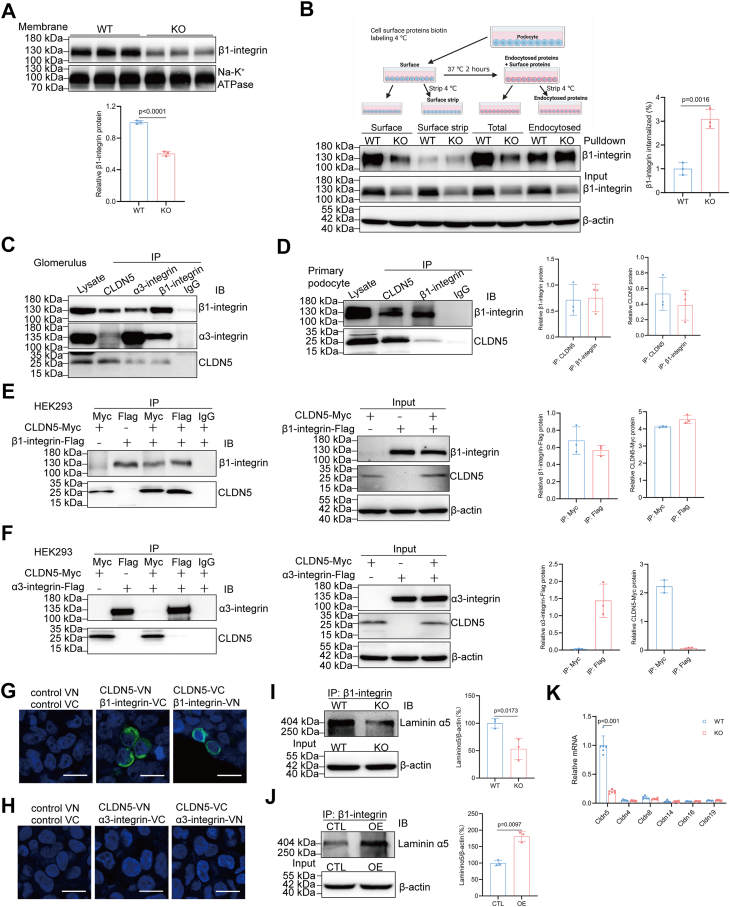
**CLDN5 forms a complex with β1-integrin to stabilize its cell surface expression**. *A*, Western blot with densitometric analysis of membrane β1-integrin in the podocytes from WT and *Cldn5*-KO mice (n = 3). *B*, pulse-chase biotinylation assay performed in WT and *Cldn5*-KO podocytes to assess β1-integrin internalization rates. Cell surface proteins were biotinylated on ice, and internalization was allowed to proceed at 37 °C for 2 h. Biotinylation from non-internalized proteins was subsequently removed chemically, while internalized biotinylated proteins were isolated using avidin-agarose and analyzed by immunoblotting. A stripping control was included to verify the removal of biotin from non-internalized proteins. To account for potential protein degradation, a non-degraded control was included after the internalization period. The internalization percentage was calculated by summing the degraded and internalized fractions and dividing by the surface fraction (n = 3). *C*, Co-IP of CLDN5, β1-integrin, and α3-integrin in isolated glomerulus to assess the interaction of endogenous CLDN5 with β1-integrin or α3-integrin. *D*, Co-IP with densitometric analysis of CLDN5 and β1-integrin in isolated podocytes to determine the interaction of endogenous CLDN5 with β1-integrin. *E*, Co-IP with densitometric analysis showing the interaction between CLDN5 and β1-integrin in doubly transfected HEK293 cells. *F*, Co-IP with densitometric analysis showing the lack of interaction between CLDN5 and α3-integrin in doubly transfected HEK293 cells. (*G* and *H*) BiFC assays were performed in doubly transfected HEK293 cells to determine interactions between CLDN5 and β1-integrin (*G*), or CLDN5 and α3-integrin (*H*). Scale bars, 20 μm. *I* and *J*, immunoprecipitation with densitometric analysis of the interaction between β1-integrin and Laminin α5 in isolated podocytes from WT and *Cldn5*-KO mice (*I*), or *Cldn5*-KO podocytes treated with lentivirus carrying either control or *Cldn5* coding sequence (*J*) (n = 3). *K*, qRT-PCR analysis of *Cldn5*, *Cldn4*, *Cldn8*, *Cldn14*, *Cldn16*, and *Cldn19* mRNA in the isolated podocytes from WT and *Cldn5*-KO mice (n = 6). For all immunoprecipitation or Co-IP analyses, antibodies used for immunoprecipitation are shown above the lanes; antibody for blot visualization is shown on the *right*. Control IPs with singly transfected cells (β1-integrin or CLDN alone) confirm specificity and exclude nonspecific binding. Whole-cell lysates (input) from singly and doubly transfected cells demonstrate expression levels of CLDNs and β1-integrin. Data are presented as mean ± SD. Statistical significance was determined by two-tailed Student’s unpaired *t* test analysis (*A*, *B*, *I*, and *J*), two-way ANOVA followed by Tukey’s post-test (*K*).

Integrins are repeatedly internalized into the endosomal system, where they are subsequently either degraded by lysosomes or recycled to the plasma membrane (26). We next tested whether the deletion of *Cldn5* in podocytes affects β1-integrin surface levels. Biotinylation experiments followed by Western blot analysis revealed a decrease in membrane β1-integrin in *Cldn5*-KO podocytes compared to wild-type cells (Fig. 4*A*). Given that the loss of CLDN5 reduces the total protein levels of β1-integrin, we next investigated whether the decrease in cell surface β1-integrin is due to a global reduction in its expression or whether CLDN5 deficiency affects β1-integrin dynamics by accelerating its internalization. We used biotin pulse-chase experiments to directly measure whether altered surface levels of β1-integrin may be related to its internalization. Cells were biotinylated at 4 °C (surface fraction), then shifted to 37 °C to allow internalization (non-degrading surface fraction) and returned to 4 °C to strip biotin from surface proteins (internalized fraction). As a control, one group was kept at 4 °C after labeling to ensure complete biotin stripping from non-internalized proteins (strip fraction). Cell lysate was collected for each step and biotinylated proteins were isolated using avidin agarose and analyzed by immunoblotting for β1-integrin. The percentage of internalization was calculated by subtracting the non-degraded surface fraction from the surface fraction, adding this result to the internalized fraction, and then dividing by the surface fraction. As shown in Figure 4*B*, CLDN5 depletion led to a significant increase in β1-integrin internalization compared to control cells after 2 h at 37 °C (Fig. 4*B*). Our data indicate that CLDN5 stabilizes β1-integrin at the cell membrane and inhibits its internalization, whereas CLDN5 deficiency leads to increased internalization of cell surface β1-integrin.

To determine if CLDN5 forms a complex with α3β1-integrin in podocytes, we first performed Co-IP of CLDN5, α3-integrin, and β1-integrin. Immunoprecipitation of glomerular or primary podocyte lysates with anti-β1-integrin antibody and immunoblotting with anti-CLDN5 antibody or immunoprecipitation of lysates with anti-CLDN5 antibody and immunoblotting with anti-β1-integrin and anti-α3-integrin antibodies demonstrated that CLDN5 co-immunoprecipitated with α3β1-integrin *in vivo* (Fig. 4, *C* and *D*). To verify further the interaction between α3β1-integrin and CLDN5, we transfected CLDN5 with α3-integrin or β1-integrin to reconstitute these proteins in HEK293 cells. By immunoprecipitating integrin or CLDN5 using either anti-CLDN5 or anti-integrin antibodies in these cells, we found that β1-integrin, but not α3-integrin, interacted directly with CLDN5 (Fig. 4, *E* and *F*). This suggests that the interaction of α3-integrin with CLDN5 *in vivo* is indirect. To further evaluate whether CLDN5 interacts with β1-integrin, we performed BiFC assay based on the fluorescent protein Venus. We fused CLDN5 and β1-integrin to the N- and C-terminal fragments of Venus, respectively, generating CLDN5-VN and β1-integrin-VC. When CLDN5-VN and β1-integrin-VC were expressed together but not individually, fluorescence was detected (Fig. 4*G*). We also generated CLDN5-VC and β1-integrin-VN and again observed fluorescence only when these fusions were expressed together (Fig. 4*G*). Thus, BiFC assays demonstrated a direct interaction between CLDN5 and β1-integrin in HEK293 cells by reconstituting Venus fluorescence, a specificity further confirmed by the lack of interaction with α3-integrin (Fig. 4*H*). Additionally, Co-IP experiments in non-podocyte cells showed no significant CLDN5–β1-integrin interaction (Fig. S3), underscoring the predominantly podocyte-specific nature of this complex, likely driven by podocyte-specific polarized architecture and expression profile.

To determine whether the physical interaction between CLDN5 and α3β1-integrin has functional significance, we investigated whether CLDN5 depletion in podocytes affects the binding of α3β1-integrin to its major ligand, laminin-α5β2γ1. Immunoprecipitation of β1-integrin yielded more laminin α5 when CLDN5 was present, but less when absent (Fig. 4*I*), indicating that depletion of CLDN5 decreases the ligand-binding of α3β1-integrin. The introduction of CLDN5 into CLDN5-deficient podocytes resulted in enhanced α3β1-integrin and laminin α5 association (Fig. 4*J*). These findings may be explained by decreased localization of β1-integrin on the cell membrane in CLDN5-deficient podocytes or by the possibility that CLDN5 deficiency impairs the binding affinity of α3β1-integrin for its extracellular matrix ligands. Investigation of other β1-integrin-binding CLDNs showed negligible basal expression of CLDN4, CLDN8, CLDN14, CLDN16, and CLDN19 and no significant compensatory upregulation upon *Cldn5* knockout in podocytes, thereby demonstrating CLDN5's predominant role at the podocyte–GBM interface (Fig. 4*K*). Together, these data provide clear evidence that CLDN5 has an important role in supporting the function of β1-integrin in podocytes.

### CLDN5 interacts with β1-integrin through its ICL and C-term domains

**Figure 5:**
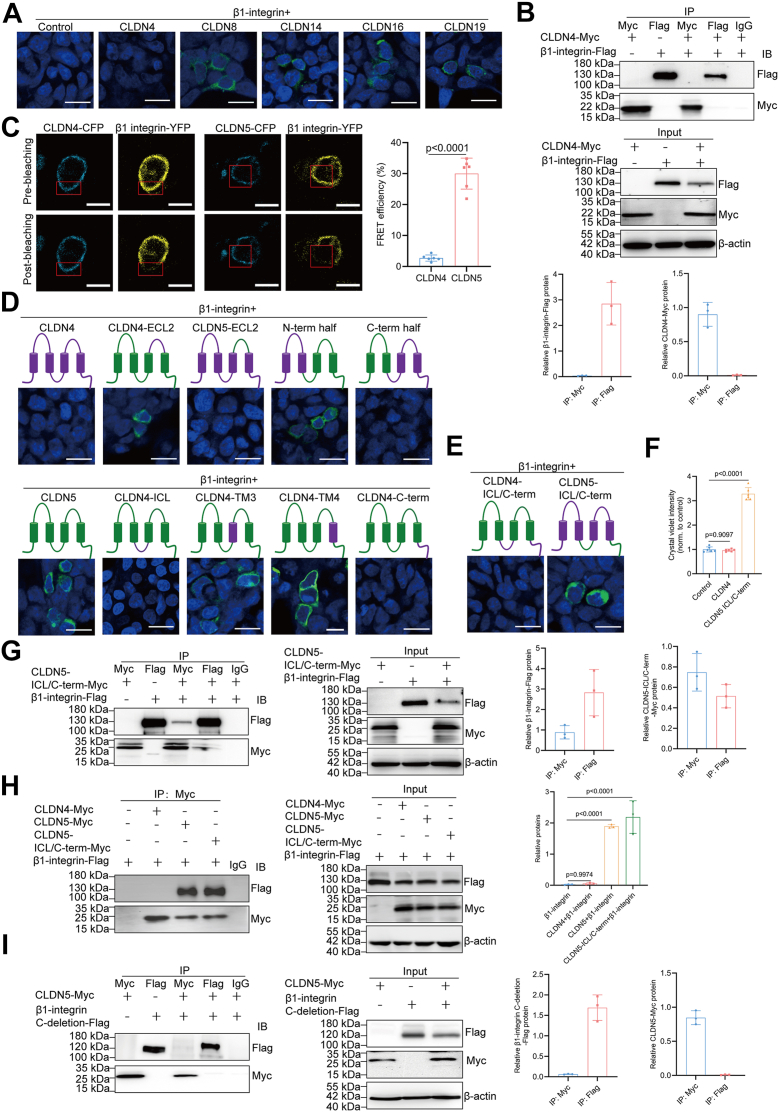
**CLDN5 interacts with β1-integrin through its ICL and C-term domains**. *A*, BiFC assay to assess the interaction between β1-integrin and CLDNs in doubly transfected HEK293 cells. Scale bars, 20 μm. *B*, Co-IP with densitometric analysis of Myc-tagged CLDN4 and Flag-tagged β1-integrin in doubly transfected HEK293 cells to determine the interaction between CLDN4 and β1-integrin. *C*, representative images showing CFP fluorescence intensity before and after photobleaching of EYFP in HEK293 T cells co-transfected with CLDN5-ECFP/β1-integrin-EYFP or CLDN4-ECFP/β1-integrin-EYFP. *Red boxes* demarcate the photobleached regions. Quantification of FRET efficiency (percentage of energy transferred from the CFP donor to the YFP acceptor, indicating the proximity of the two proteins) reveals a significantly higher interaction between CLDN5 and β1-integrin compared to CLDN4 and β1-integrin (CLDN5: 30.00% FRET efficiency; CLDN4: 2.48% FRET efficiency). Scale bars, 10 μm. *D*, BiFC assay to assess the interaction between β1-integrin and CLDN chimera in doubly transfected HEK293 cells. CLDN4-ECL2 represents a CLDN5 chimera containing the extracellular loop 2 (ECL2) of CLDN4; CLDN5-ECL2 represents a CLDN4 chimera containing the ECL2 of CLDN5; N-term half represents a CLDN4 chimera containing N-term, TM1, ECL1, and TM2 of CLDN4 fused with ICL, TM3, ECL2, TM4, and C-term of CLDN5; C-term half represents a CLDN4 chimera containing N-term, TM1, ECL1, and TM2 of CLDN5 fused with ICL, TM3, ECL2, TM4, and C-term of CLDN4. CLDN4-ICL represents a CLDN5 chimera containing the intracellular loop (ICL) of CLDN4; CLDN4-TM3 represents a CLDN5 chimera containing the third transmembrane domain (TM3) of CLDN4; CLDN4-TM4 represents a CLDN5 chimera containing the fourth transmembrane domain (TM4) of CLDN4; and CLDN4-C-term represents a CLDN5 chimera containing the C-terminal tail (C-term) of CLDN4. Scale bars, 20 μm. *E*, BiFC assay to determine the requirement of ICL and C-term for the interaction between β1-integrin and CLDN5 in doubly transfected HEK293 cells. CLDN4-ICL/C-term represents a CLDN5 chimera containing the ICL and C-term of CLDN4, while CLDN5-ICL/C-term represents a CLDN4 chimera containing the ICL and C-term of CLDN5. Scale bars, 20 μm. *F*, relative numbers of adherent cells determined by crystal violet staining in the isolated *Cldn5*-KO podocytes treated with lentivirus carrying either control, CLDN4, or CLDN4 chimera containing the ICL and C-term of CLDN5 sequences (n = 6). *G*, Co-IP with densitometric analysis of Myc-tagged CLDN4 chimera containing the ICL and C-term of CLDN5 and Flag-tagged β1-integrin to confirm the requirement of ICL and C-term for the interaction between β1-integrin and CLDN5 in doubly transfected HEK293 cells. *H*, Co-IP with densitometric analysis of CLDN and β1-integrin in doubly transfected HEK293 cells to detect the interaction between different CLDN and β1-integrin. *I*, Co-IP with densitometric analysis of CLDN5 and β1-integrin with C-terminal deletion in doubly transfected HEK293 cells to assess the interaction between CLDN5 and truncated β1-integrin. For all immunoprecipitation or Co-IP analyses, antibodies used for immunoprecipitation are shown above the lanes; antibody for blot visualization is shown on the *right*. Control IPs with singly transfected cells (β1-integrin or CLDN alone) confirm specificity and exclude nonspecific binding. Whole-cell lysates (input) from singly and doubly transfected cells demonstrate expression levels of CLDNs and β1-integrin. Data are presented as mean ± SD. Statistical significance was determined by two-tailed Student’s unpaired *t* test (*C*), one-way ANOVA followed by Tukey’s post-test (*F* and *H*).

As this is the first observation of CLDN5 binding to β1-integrin, we next sought to identify the binding domain of CLDN5 that mediates the interaction between these two proteins. The BiFC study demonstrated that CLDN8, CLDN14, CLDN16, and CLDN19, but not CLDN4, bind to β1-integrin (Fig. 5*A*). Since a previous study reported that the tight junction-unintegrated CLDN4 binds β1-integrin in T24 cells (15), we further investigated whether CLDN4 interacts with β1-integrin in HEK293 cells jhkjhj using Co-IP. In doubly transfected cells, the β1-integrin antibody did not precipitate CLDN4, nor did the CLDN4 antibody precipitate β1-integrin (Fig. 5*B*). Complementary acceptor photobleaching Förster Resonance Energy Transfer (FRET) experiments, where CLDN4 or CLDN5 were fused to ECFP and β1-integrin to EYFP in HEK293 T cells, demonstrated a significantly higher FRET efficiency for CLDN5/β1-integrin (30.00%) compared to CLDN4/β1-integrin (2.48%), providing further evidence for a specific interaction between CLDN5 and β1-integrin (Fig. 5*C*). Thus, BiFC, Co-IP, and FRET analyses failed to demonstrate an interaction between β1-integrin and CLDN4, we constructed a panel of chimeric CLDN4/CLDN5 fusion proteins to map the CLDN5 region involved in its interaction with β1-integrin. The chimeras were expressed in HEK293 cells and screened for their capability to interact with β1-integrin *via* BiFC assay. Nevertheless, all chimeric constructs retained their ability to localize to the cell membrane when expressed with a C-terminal mCherry tag in HEK293 cells (Fig. S4), confirming the successful expression and proper subcellular targeting of these engineered proteins. Previous studies have implicated the extracellular loop 2 (ECL2) domain as critical for mediating interactions between tetraspanins and integrins. Contrary to this expectation, our results revealed that a CLDN5 chimeric protein, in which the native ECL2 was replaced with the corresponding domain from CLDN4, still maintained its ability to interact with β1-integrin (Fig. 5*D*). Further, β1-integrin couldn’t bind to CLDN4 chimera with a substituted ECL2 from CLDN5 (Fig. 5*D*). These data indicated ECL2 domain of CLDN5 is not necessary for its interaction with β1-integrin.

Chimeric CLDN acquired β1-integrin binding ability when the N-term half (N-term, TM1, ECL1, and TM2) of CLDN4 was fused with the C-term half (ICL, TM3, ECL2, TM4, and C-term) of CLDN5 (Fig. 5*D*). However, β1-integrin did not bind a chimera with the N-term half of CLDN5 fused to the C-term half of CLDN4 (Fig. 5*D*). These results indicated the presence of a β1-integrin binding region in the C-term half of CLDN5. By individually replacing the ICL, TM3, TM4, and C-term of CLDN5 with corresponding regions of CLDN4, it was found that only the replacements of TM3 and TM4 still retained the ability to interact with β1-integrin (Fig. 5*D*). This suggests that both the ICL and C-term are required for the interaction between CLDN5 and β1-integrin. As shown in Figure 5*E*, chimeras exchanging the ICL and C-term regions of CLDN4 with that of CLDN5 confer the ability to bind to β1-integrin (Fig. 5*E*). In contrast, the exchange of ICL and C-term regions of CLDN5 by that of CLDN4 was sufficient to diminish β1-integrin-CLDN5 interaction (Fig. 5*E*). Further, the expression of wild-type CLDN4 fails to rescue the adhesion ability of CLDN5-deficient podocytes (Fig. 5*F*). In contrast, the reconstitution of the CLDN5 ICL and C-term regions within the CLDN4/5 chimera rescued adhesion ability of CLDN5-deficient podocytes (Fig. 5*F*). The direct interaction of the ICL and C-term region of CLDN5 with β1-integrin was also confirmed by Co-IP experiments (Fig. 5*G*). To further validate the differential interactions of claudin proteins with β1-integrin under identical conditions, we transfected cells with β1-integrin-Flag alone or in combination with CLDN4-Myc, CLDN5-Myc, or the chimera CLDN5-ICL/C-term-Myc, followed by Myc immunoprecipitation and Western blotting for Flag and Myc. Robust Co-IP of β1-integrin was observed with CLDN5-Myc and the chimera, but not with CLDN4-Myc, confirming the interaction of CLDN5 with β1-integrin and the involvement of its ICL and C-terminal regions in this binding (Fig. 5*H*). These results collectively demonstrate that the interaction between CLDN5 and β1-integrin is mediated primarily through the ICL and the C-term regions of CLDN5. Given that both the ICL and the C-term regions of CLDN5 are localized intracellularly, we speculated that the intracellular domain of β1-integrin mediates the interaction between CLDN5 and β1-integrin. To test this, we constructed an β1-integrin truncation mutant lacking the intracellular domain and co-expressed it with CLDN5 in HEK293 cells. The results showed that CLDN5 failed to interact with the β1-integrin truncation mutant (Fig. 5*I*), indicating that the intracellular domain of β1-integrin is essential for its interaction with CLDN5. This finding confirms our hypothesis that the interaction between CLDN5 and β1-integrin occurs in the intracellular space, specifically involving both the ICL and C-terminal regions of CLDN5 and the intracellular domain of β1-integrin.

### CLDN5 reinforces podocyte adhesion *in vivo*

**Figure 6:**
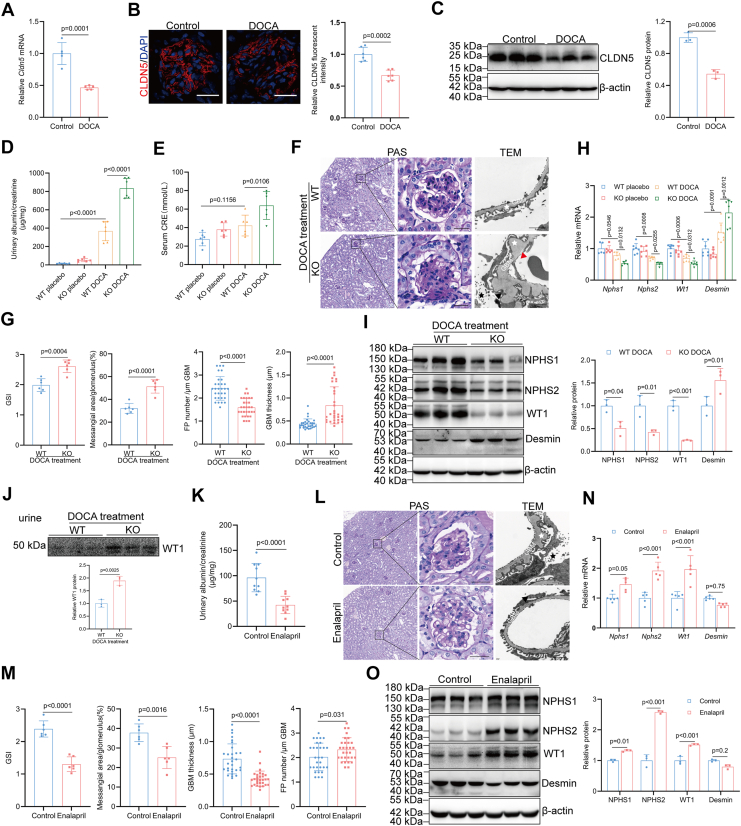
**CLDN5 deficiency in podocytes exacerbates hypertensive nephropathy**. *A–C*, qRT-PCR analysis (*A*, n = 5), immunofluorescence (*B*, n = 6), and Western blot with densitometric analysis (*C*, n = 3) of CLDN5 in glomerulus from mice subjected to DOCA/high-salt-induced hypertension. Scale bars, 20 μm. *D*, Albumin-to-creatinine ratio (μg/mg) in urine from WT and *Cldn5*-KO mice subjected to DOCA/high-salt-induced hypertension (n = 6). *E*, serum creatinine levels of WT and *Cldn5*-KO mice subjected to DOCA/high-salt-induced hypertension (n = 6). *F* and *G*, morphological examinations of glomerular changes by PAS staining (n = 6 biologically independent animals, 10 glomeruli per mouse were analyzed) and TEM analyses (n = 3 biologically independent animals, 10 images per group) in kidneys from WT and *Cldn5*-KO mice subjected to DOCA/high-salt-induced hypertension. *White stars* denote GBM spikes, *red triangles* indicate GBM splitting, *black stars* denote podocyte microvilli, and *black triangles* highlight foot process effacement. Scale bars: 20 μm for PAS staining images and 2 μm for TEM images. *H*, expression of *Nphs1*, *Nphs2*, *Wt1*, and *Desmin* analyzed by qRT-PCR in glomerulus from WT and *Cldn5*-KO mice subjected to DOCA/high-salt-induced hypertension (n = 5). *I*, Western blot with densitometric analysis of NPHS1, NPHS2, WT1, and Desmin in glomerulus from WT and *Cldn5*-KO mice subjected to DOCA/high-salt-induced hypertension (n = 3). *J*, Western blot with densitometric analysis of WT1 in urine from WT and *Cldn5*-KO mice subjected to DOCA/high-salt-induced hypertension (n = 3). *K*, albumin-to-creatinine ratio (μg/mg) in urine from 8-month-old *Cldn5*-KO mice treated with or without enalapril (n = 10). *L and M*, morphological examinations of glomerular changes by PAS staining (n = 6 biologically independent animals, 10 glomeruli per mouse were analyzed) and TEM analyses (n = 3 biologically independent animals, 10 images per group) in kidneys from 8-month-old *Cldn5*-KO mice with or without enalapril treatment. *White stars* indicate GBM spikes, *black stars* denote podocyte microvilli, and *black triangles* highlight foot process effacement. Scale bars: 20 μm for PAS staining images and 2 μm for TEM images. *N*, qRT-PCR analysis of *Nphs1*, *Nphs2*, *Wt1*, and *Desmin* in glomerulus from 8-month-old *Cldn5*-KO mice with or without enalapril treatment (n = 5). *O*, Western blot with densitometric analysis of NPHS1, NPHS2, WT1, and Desmin in glomerulus from *Cldn5*-KO mice with or without enalapril treatment (n = 3). Data are presented as mean ± SD. Statistical significance was determined by two-tailed Student’s unpaired *t* test (*A*, *B*, *C*, *G*, *J*, *K*, and *M*), one-way ANOVA followed by Tukey’s post-test (*D*, *E*, and *H*), two-way ANOVA followed by Tukey’s post-test (*I*, *N*, and *O*).

Given the impaired podocyte adhesion to the GBM observed in *Cldn5*-deficient cells, we investigated whether the absence of podocyte CLDN5 would render mice more susceptible to increased transcapillary filtration pressure. We then focused on the role of CLDN5 in hypertensive renal injury and established an *in vivo* hypertensive renal injury model in wild-type and *Cldn5*-KO mice. This was achieved by subcutaneously implanting 21-days release pellets containing 50 mg of deoxycorticosterone acetate (DOCA) following right nephrectomy and providing saline as drinking water, which effectively overcame hypertension resistance in the salt-resistant C57BL/6 strain (27). Although blood pressure increased significantly after implanting DOCA for 21 consecutive days, there was no significant difference between wild-type and *Cldn5*-KO mice (Fig. S5*A*). As shown in Figure 6, *A*–*C*, expression of CLDN5 in the glomerulus was significantly lower in hypertensive nephropathy, indicating that CLDN5 was responsive to hypertensive renal injury. The urine albumin-creatinine ratio (uACR) and serum creatinine levels of *Cldn5*-KO mice were significantly higher than that of wild-type mice (Fig. 6, *D* and *E*). Periodic Acid-Schiff (PAS) staining and Masson Thichrome staining (MTS) indicated that glomerular segmental sclerosis was more severe in *Cldn5*-KO mice compared to wild-type mice after hypertensive renal injury (Fig. 6, *F* and *G*, S5*B*). qRT-PCR, Western blot, and immunofluorescence staining revealed increased expression of the podocyte injury indicator desmin and reduced expression of the key podocyte markers, including nephrin (NPHS1), podocin (NPHS2), and Wilms tumor 1 (WT1) in hypertensive *Cldn5*-KO mice compared to hypertensive wild-type mice (Fig. 6, *H* and *I*, S5*C*), further confirming the aggravation of podocyte damage. Transmission electron microscopy analysis revealed that *Cldn5* knockout significantly exacerbated foot process effacement, reduced filtration slit density, and led to a disorganized and thickened GBM with local protrusions in hypertensive mice (Fig. 6, *F* and *G*). Western blot analysis of the expression of WT1 in the 24-h urine sample confirmed the urinary loss of podocytes in hypertensive *Cldn5*-KO mice (Fig. 6*J*). These results collectively indicate that knockout of *Cldn5* exacerbates DOCA-induced renal injury without affecting DOCA-induced elevation in blood pressure. Conversely, administering the angiotensin-converting enzyme (ACE) inhibitor enalapril to reduce both systemic and intraglomerular blood pressure protected 8-month-old *Cldn5*-KO mice from renal injury. This protection was evidenced by reduced proteinuria (Fig. 6*K*) and improvements in glomerulosclerosis, as demonstrated through PAS (Fig. 6, *L* and *M*), MTS (Fig. S5*D*), transmission electron microscopy (Fig. 6, *L* and *M*), immunofluorescence analyses (Fig. S5*E*), qRT-PCR (Fig. 6*N*), and Western blot (Fig. 6*O*). Collectively, these findings indicate that podocyte adhesion to the GBM promoted by CLDN5 is important for the stability and function of podocytes, especially during mechanical stress induced by high intraglomerular capillary pressure.

**Figure 7:**
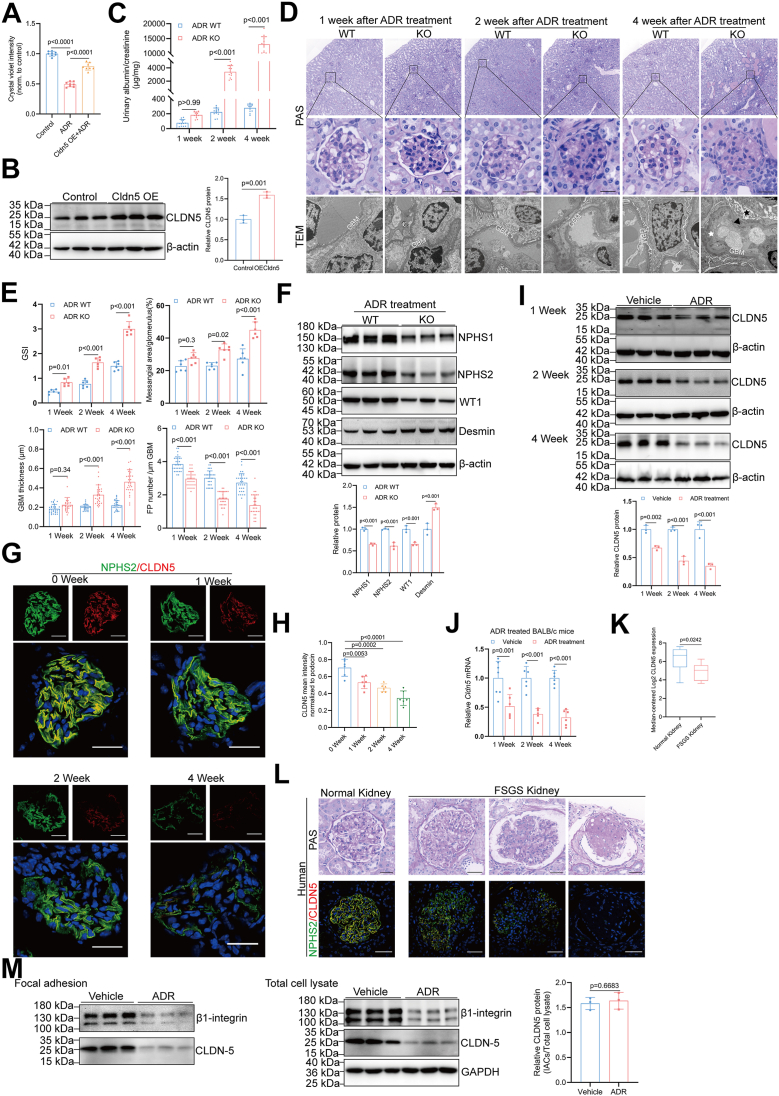
**Podocyte CLDN5 deficiency renders C57BL/6 mice susceptible to adriamycin-induced nephropathy**. *A*, relative number of adherent cells in primary podocytes treated with lentivirus carrying either a control or CLDN5 coding sequence for 24 h followed by 12-h treatment with 10 μM adriamycin as determined by crystal violet staining (n = 8). *B*, Western blot analysis and densitometric quantification of CLDN5 in primary podocytes transduced with lentivirus carrying either a control sequence or the CLDN5 coding sequence (n = 3). *C*, albumin-to-creatinine ratio (μg/mg) in urine from WT and *Cldn5*-KO C57BL/6 mice following adriamycin treatment (n = 12). *D* and *E*, morphological examinations of glomerular changes by PAS staining (n = 6 biologically independent animals, 10 glomeruli per mouse were analyzed) and TEM analyses (n = 3 biologically independent animals, 10 images per group) in kidneys from WT and *Cldn5*-KO C57BL/6 mice following adriamycin treatment. *White stars* indicate GBM spikes, *black stars* denote podocyte microvilli, and *black triangles* highlight foot process effacement. Scale bars: 20 μm for PAS staining images and 2 μm for TEM images. *F*, Western blot with densitometric analysis of NPHS1, NPHS2, WT1, and Desmin in glomerulus from WT or *Cldn5*-KO C57BL/6 mice following adriamycin treatment (n = 3). *G* and *H*, immunofluorescence staining (*G*) and quantitative analysis (*H*) of NPHS2 (*green*) and CLDN5 (*red*) in kidneys from BALB/c mice following adriamycin treatment (n = 6). Scale bars, 20 μm. *I*, Western blot with densitometric analysis of CLDN5 in glomerulus from BALB/c mice following adriamycin treatment (n = 3). *J*, qRT-PCR analysis of *Cldn5* mRNA abundance in glomerulus from BALB/c mice following adriamycin treatment (n = 6). *K*, Nephroseq analysis comparing *CLDN5* expression level in normal kidney (n = 9) vs. FSGS kidney (n = 8) from Hodgin FSGS glomerulus dataset. Whiskers represent the 10th/90th percentile values as precomputed within Nephroseq. The *box* represents the middle quartiles, the lines indicate the median, and the whiskers plots depict the maximum and minimum values of each group. Nephroseq (www.nephroseq.org, 04/2025, University of Michigan, Ann Arbor, MI) was used for analysis and visualization. *L*, PAS staining and immunofluorescence of CLDN5 (*red*) and NPHS2 (*green*) in normal and FSGS kidneys. CLDN5 expression displays a heterogeneous pattern: globally sclerotic glomeruli show minimal expression, segmentally sclerotic glomeruli exhibit enhanced staining in nonsclerotic areas, and fully preserved glomeruli demonstrate overall increased CLDN5 staining. Scale bars, 50 μm. *M*, Western blot and densitometric analysis of CLDN5 in adhesion complexes and total cell lysate of primary podocytes isolated from BALB/c mice following vehicle or adriamycin treatment (n = 3). Data are presented as mean ± SD. Statistical significance was determined by one-way ANOVA followed by Tukey’s post-test (*A* and *H*), two-tailed Student’s unpaired *t* test (*B*, *K*, and *M*), two-way ANOVA followed by Tukey’s post-test (*C*, *E*, *F*, *I*, and *J*).

We subsequently explored the role of CLDN5 in adriamycin-induced podocyte injury, a condition known to disrupt podocyte adhesion by decreasing β1-integrin expression and compromising focal adhesion dynamics (28). *In vitro* adhesion assays revealed that adriamycin treatment significantly impaired podocyte attachment, an effect that was rescued by CLDN5 transfection (Fig. 7, *A* and *B*). While C57BL/6 mice are inherently resistant to adriamycin-induced nephropathy, male *Cldn5*-KO mice on this genetic background exhibited severe proteinuria, pronounced foot process effacement, significant podocyte loss, and marked glomerulosclerosis following adriamycin treatment (Fig. 7, *C*–*F* and S6). Similarly, female *Cldn5*-KO C57BL/6 mice also exhibited susceptibility to adriamycin-induced nephropathy, although the extent of glomerular injury was milder compared to their male counterparts (Fig. S7). These findings suggest that podocyte CLDN5 deletion converts the C57BL/6 strain from being adriamycin nephropathy-resistant to -susceptible. In adriamycin-treated BALB/c mice with podocytopathy (Fig. S8), glomerular CLDN5 expression (normalized to podocin) was significantly reduced, as validated by immunofluorescence, Western blot, and qRT-PCR (Fig. 7, *G*–*J*). Consistently, Nephroseq analysis demonstrated reduced glomerular CLDN5 mRNA in FSGS patients *versus* healthy controls (Fig. 7*K*), while immunofluorescence of FSGS biopsies confirmed progressive podocyte CLDN5 loss that correlated with histopathological injury severity (Fig. 7*L*). These data demonstrate that reduced podocyte CLDN5 is a shared feature of podocytopathy in both adriamycin-induced injury and human FSGS. Specifically, the stable ratio of CLDN5 within isolated adhesion complexes indicates that the observed pathological reduction in CLDN5 results from decreased expression levels rather than a dynamic redistribution within podocytes from BALB/c mice following adriamycin treatment (Fig. 7*M*).

In nephropathy-susceptible BALB/c mice, *Cldn5* knockout triggered early-onset proteinuria detectable by postnatal day 14 (P14) (Fig. S9*A*). Morphological analyses demonstrated progressive renal injury, characterized by substantial foot process effacement by P7, segmental GBM splitting by P14, and severe glomerulosclerosis evident by P21 (Fig. S9, *B* and *C*). Immunofluorescence analysis demonstrated concurrent molecular evidence of podocyte injury, with upregulated desmin and downregulated NPHS1, NPHS2, and WT1 expression at P14 and P21 (Fig. S9, *D*–*F*), indicative of ongoing podocyte depletion. Using AAVs to express either wild-type CLDN5 or the CLDN5-CLDN4 chimera in the kidneys of *Cldn5*-KO mice, we found that wild-type CLDN5 rescued albuminuria, glomerular damage, and podocyte morphology while the chimera lacking the β1-integrin binding motif did not (Fig. S10), confirming CLDN5's role in stabilizing β1-integrin and maintaining kidney function. Notably, BALB/c glomeruli exhibited an 52% decrease in *Cldn5* mRNA expression and a 59.4% reduction in CLDN5 protein levels compared to C57BL/6, as determined by qRT-PCR, Western blot, and confocal imaging (Fig. S9, *G*–*I*). This reduced expression may contribute to their increased nephropathy susceptibility.

## Discussion

Our findings reveal that CLDN5 serves as a novel regulator of podocyte adhesion by interacting with β1-integrin. This interaction enhances cellular anchoring, equipping podocytes with increased resistance against mechanical stresses imposed by primary urine filtration and repetitive stretching forces. The absence of CLDN5 may therefore increase podocyte vulnerability to mechanical stress and promote detachment.

Emerging evidence indicates that the CLDN protein family, while best characterized for its tight junction functions, participates in cell-matrix interactions. For instance, CLDN7 has been shown to interact with β1-integrin in lung cells and α2-integrin in intestinal cells, both of which facilitate cell-matrix adhesion independent of their roles in tight junctions (11, 12). Similarly, CLDN11 forms a complex with OAP-1 and β1-integrin to regulate oligodendrocyte proliferation and migration, further highlighting the non-junctional functions of CLDNs in cell adhesion (13). Additionally, CLDN2 has been reported to enhance the surface expression of integrin complexes such as α2β1-and α5β1-integrin in breast cancer cells, promoting adhesion to ECM components like fibronectin and type IV collagen (14). These data suggest that cell signaling through CLDN-integrin pathway may represent a more universal mechanism. However, additional data is required to bolster this interpretation. Earlier studies using sucrose gradient centrifugation of glomerular lysates and antibody-based immunoelectron microscopy have established CLDN5 localization within podocyte cell bodies and along the GBM of wild-type mouse kidneys (19, 22). Here, employing Western Blot analysis of enriched adhesion complexes, as well as super-resolution expansion microscopy and detailed immunolocalization, we could furthermore demonstrate that CLDN5 is enriched within the podocyte adhesion complex. Although the mechanisms by which CLDN5 achieves this subcellular localization were not addressed, this precise localization hints at a direct involvement of CLDN5 in modulating podocyte-ECM interactions, as supported by our functional data showing that deletion of *Cldn5* in podocytes impairs adhesion and spreading on various ECM components and introduces vulnerability to mechanical stress. The presence of CLDN5 at both the apical and basal domains underscores its complex and multifaceted role within the intricate machinery of podocytes.

Our mechanistic investigations revealed that CLDN5 enhances podocyte adhesion by stabilizing β1-integrin expression and localization at the plasma membrane. Although β1-integrin mRNA levels and activation status remained unchanged in *Cldn5-*KO podocytes, the loss of CLDN5 resulted in a reduction of β1-integrin protein expression. Notably, *Cldn5* knockdown in primary podocytes directly decreased β1-integrin levels and impaired cell adhesion, effects that were partially rescued by ectopic expression of β1-integrin. These findings strongly suggest that CLDN5’s role in podocyte adhesion is, at least in part, mediated through its regulation of β1-integrin protein level. We found that β1-integrin degradation was accelerated in CLDN5-deficient podocytes, a process sensitive to proteasome inhibition but not lysosomal inhibition. Furthermore, CLDN5 ablation led to significantly elevated levels of ubiquitinated β1-integrin. This implicates the ubiquitin-proteasome system as the primary pathway for β1-integrin degradation in the absence of CLDN5. The ubiquitin-proteasome system has been proven to regulate key podocyte functions by maintaining the glomerular cell-specific proteome (29). The E3 ubiquitin ligase UBR4 has been shown to control polyubiquitylation and stability of podocin and its *Caenorhabditis elegans* orthologue MEC-2 (30). Similarly, mechanisms such as CIN85-mediated ubiquitination of nephrin and synaptopodin's interaction with the E3 ligase CBL have been shown to influence podocyte stability and cytoskeletal dynamics (31, 32, 33). WT1 is specifically targeted for ubiquitin-mediated proteasomal degradation by β-catenin (34). UBD, a ubiquitin-like protein modifier that targets proteins for proteasomal degradation, interacts with APOL1 to modulate APOL1 risk variant-mediated cell death (35). Our findings extend this knowledge by demonstrating that CLDN5 plays a crucial role in modulating β1-integrin stability through a mechanism involving the ubiquitin-proteasome system.

Mass spectrometry analysis of β1-integrin antibody IP products identified the E3 ligase HUWE1 as a regulator of β1-integrin stability. Given that the intracellular domain of β1-integrin interacts with both CLDN5 and HUWE1, we speculate that the interaction between CLDN5 and β1-integrin conceals the HUWE1 binding sites, making them unavailable for HUWE1 binding and thereby preventing β1-integrin ubiquitination and subsequent proteasomal degradation. However, the lack of high-resolution structural information precludes a definitive distinction between direct steric hindrance and conformational effects in the CLDN5-β1-integrin interaction; future studies will therefore focus on elucidating specific binding interfaces and structural dynamics to clarify its role in regulating β1-integrin stability. Intriguingly, while no direct HUWE1-β1-integrin interaction was observed in wild-type podocytes under basal conditions, HUWE1 nonetheless exerted downstream suppression of β1-integrin expression, suggesting the contribution of HUWE1-dependent influence on intermediate cellular proteins or signaling pathways as indirect means to control β1-integrin stability. This functional interaction between HUWE1 and β1-integrin has not been described previously, although it should be noted that HUWE1 (EEL-1 in *C*. *elegans*) has been reported to regulate cellular adhesion and ECM receptor dynamics during *C*. *elegans* development (36). In addition, we observed that CLDN5 restrains β1-integrin endocytosis, and its deficiency promotes β1-integrin internalization, thereby reducing cell surface β1-integrin available for cell matrix adhesion. This confirms CLDN5’s essential role in maintaining β1-integrin surface localization in podocytes. While the precise molecular mechanism remains to be determined, it may involve direct interaction with β1-integrin that masks internalization signals, association with membrane trafficking machinery components that stabilize surface expression, or participation in specialized membrane domains that sequester β1-integrin away from endocytic pathways.

BiFC, Co-IP, and FRET assays confirmed a direct physical interaction between CLDN5 and β1-integrin. Domain mapping further identified the ICL and C-terminal regions of CLDN5 as essential for binding β1-integrin. CLDNs share convergent structural topologies, consisting of an intracellular N-term, four α-helical TM segments, two ECL segments, one ICL segment, and an intracellular C-term (37). ECL1 exhibits significant variation in the position and number of charged amino acids contingent on the CLDN subtype and serves to form paracellular barriers or pores for selective ions and solutes between neighbouring cells (37). ECL2 is involved in both the binding of *Clostridium perfringens* enterotoxin (CPE) and the trans-interaction between the plasma membranes of opposing cells (37, 38, 39, 40). Both ECL1 and ECL2 of CLDN19 have been proved to be involved in the interaction with CPE (40). The multimers formed by CLDNs through cis- and trans-interactions *via* their TM and ECL domains contribute to the distinct adhesion and paracellular permeation properties of each tight junction strand (41). The C-term region of many CLDN subtypes contains specific sequence motifs, such as PDZ domain-binding motifs and phosphorylation consensus sites, which function as platforms for receiving or propagating a variety of intracellular signals (42, 43, 44). While other domains have been well studied, however, the ICL of CLDNs remain less well understood. Although previous studies implicating the ECL2 domain in mediating interactions between tetraspanins and integrins (45), our study found that the ECL2 domain of CLDN5 is not necessary for its interaction with β1-integrin. Indeed, our BiFC screening and subsequent chimeric experiments revealed that both the ICL and the C-terminal tail of CLDN5 are involved in the interaction with β1-integrin. These results highlight the functional significance of the ICL of CLDNs, extending beyond the well-characterized extracellular and transmembrane domains. Future studies should aim to map the precise binding interface and elucidate the detailed molecular mechanisms by which the ICL and C-term regions of CLDN5 interact with β1-integrin and how this interaction is regulated under physiological and pathological conditions.

In addition to its molecular and cellular functions, our study provides compelling *in vivo* evidence for the protective role of CLDN5 in preserving podocyte adhesion capacity when faced with the mechanical challenges of its environment. In a hypertensive renal injury model, *Cldn5*-KO mice exhibited significantly exacerbated proteinuria and glomerulosclerosis despite comparable blood pressure elevation to wild-type mice. The observation that ACE inhibitor treatment rescued renal injury in *Cldn5*-KO mice further supports the notion that CLDN5 protects podocytes from high intraglomerular capillary pressure. Our findings align with previous research on other tetraspanins, such as CD151, which also regulate integrin-mediated adhesion of podocytes. CD151 enhances α3β1-integrin-mediated adhesion in podocytes by directly binding to α3-integrin (6), whereas CLDN5 strengthens this adhesion by directly binding to β1-integrin. Although α3β1-integrin is the primary ECM receptor required for podocyte attachment, the β1-integrin subunit can associate with several α subunits, and multiple αβ1 integrins contribute to the interaction with the GBM. Consistent with this, mice with podocyte-specific *Cldn5* deletion on a C57BL/6 background develop obvious proteinuria by 12 weeks of age (23), whereas *Cd151* knockout mice on the same background show histologically normal kidneys and no signs of proteinuria at the same age compared to age-matched controls (8). This is likely due to the remaining αβ1 integrins in the *Cd151* knockout model being sufficient to support proper podocyte adhesion. In a parallel model, we observed that CLDN5 deficiency renders the normally resistant C57BL/6 mouse strain susceptible to adriamycin-induced nephropathy (46, 47, 48), with severe podocyte injury and glomerular damage. Notably, reduced CLDN5 expression is observed in both adriamycin-treated BALB/c mice and human FSGS biopsies, correlating with the severity of glomerular injury. These observations suggest that CLDN5 downregulation is a common feature of podocytopathy and may contribute to disease progression. The susceptibility of *Cldn5*-KO mice to early-onset proteinuria and progressive renal injury in the nephropathy-prone BALB/c background further supports the protective role of CLDN5. The lower baseline expression of CLDN5 in BALB/c mice compared to C57BL/6 may underlie their inherent susceptibility to glomerular injury, a hypothesis that warrants further investigation.

Together, our findings demonstrate that CLDN5 strengthens podocyte adhesion to the GBM—a function distinct from its canonical role in tight junctions. This establishes CLDN5 as a key regulator of cell-matrix adhesion and mechanoprotection in podocytes, positioning it as a promising therapeutic target for preserving glomerular integrity in various diseases associated with glomerular hyperfiltration. Future studies should explore whether CLDN5-mediated integrin regulation represents a broader mechanoprotective mechanism in other mechanically stressed epithelia.

## Experimental procedures

### Chemicals, antibodies, plasmids, and cell lines

The chemicals and antibodies utilized in this study are detailed in the Tables S1 and S2.

Full-length cDNAs of mouse *Huwe1* (NM_021523), *β1-integrin* (NM_010578), *Cldn5* (NM_013805), and *α3-integrin* (NM_013565) were synthesized and cloned into the pLVX-mCherry vector by Sango Biotechnology. pBiFC-VN155 (I152 L) and pBiFC-VC155 vectors were kindly provided by Chang-Deng Hu (Addgene plasmids #27097 and #22011). Full-length cDNAs of human *CLDN5* (NM_001130861.1), *β1-integrin* (NM_002211.4), *CLDN4* (NM_001305.5), *CLDN8* (NM_199328.3), *CLDN14* (NM_144492.3), *CLDN16* (NM_006580.4), and *CLDN19* (NM_148960.3) were synthesized and cloned into the pBiFC-VN155 (I152L) vector by Sango Biotechnology. Full-length cDNAs of human CLDN5 and β1-integrin were also cloned into the pBiFC-VC155 vector. CLDN chimera constructs (N-term half, C-term half, CLDN4-ECL2, CLDN5-ECL2, CLDN4-ICL, CLDN4-TM3, CLDN4-TM4, CLDN4-C-term, CLDN4-ICL/C-term, CLDN5-ICL/C-term) were synthesized and cloned into the pBiFC-VN155 (I152L) vector by Beijing Tsingke Biotech (China). The coding sequence of human *β1-integrin* (NM_002211.4) was synthesized and cloned into the pcDNA3.1-Flag vector by Sango Biotechnology. The coding sequence of the CLDN chimera (CLDN5-ICL/C-term) was cloned into the pcDNA3.1-myc vector by Sango Biotechnology. Site-directed mutagenesis of the mouse *β1-integrin* gene was performed using a PCR-based mutagenesis method (NEB). Human CLDN4, CLDN5, or the CLDN chimera constructs were amplified by PCR and cloned into the pLVX-mCherry vector. To generate a truncated β1-integrin expression plasmid, the C-terminal region of β1-integrin, located in the cytosol, was deleted using PCR-based mutagenesis (with pLVX-ITGB1 as the template). Full-length cDNAs of human CLDN5 and CLDN4 were cloned into the pAmCyan1 vector. Full-length cDNAs of human β1-integrin was cloned into the pEYFP vector. The constructs were verified by DNA sequencing and by production of the correct size polypeptides following transfection and immunoblotting. Primers used for cloning are listed in Table S3. All constructs were verified by sequencing at Sangon Biotech.

HEK293 cells and 3T3-L1 cells were obtained from ATCC and cultured according to the distributor’s recommendations.

### Human kidney samples

Kidney biopsy samples were obtained from the patients with diagnosed FSGS in the Second Hospital of Shandong University. Whereas control samples were obtained from para-tumor tissues in patients who underwent surgical removal of renal carcinoma. There was no statistical difference in the general information of patients (gender, age) between the two groups. Informed written consent was obtained from all participants or next of kin. The research activities being reported are consistent with the Principles of the Declaration of Istanbul as outlined in the Declaration of Istanbul on Organ Trafficking and Transplant Tourism. The studies involving human samples were conducted in compliance with the Declaration of Helsinki and approved by the Medical Ethics Committee at the Second Hospital, Shandong University, China (reference No. KYLL2024197).

### Animals

All animal studies were approved by the Animal Ethics Committee of Binzhou Medical University and conducted in accordance with the National Institutes of Health Guide for the Care and Use of Laboratory Animals. Mice were housed in a standard environment with a 12-h light/dark cycle, a temperature of 22 to 25 °C, and 40 to 60% humidity. They were kept in a pathogen-free, climate-controlled facility and provided with food and water ad libitum.

C57BL/6J mice were obtained from Vital River Laboratory (China). The podocyte-specific *Cldn5* knockout mice (*Nphs2*-Cre^+/−^/*Cldn5*^loxP/loxP^, referred to as *Cldn5*-KO) were generated as previously described (23). All experiments used mice on a C57BL/6 background unless otherwise noted. To transfer the *Cldn5*-flox allele to the BALB/c genetic background, *Cldn5*^flox/flox^; *Nphs2*-Cre^+/−^ mice on a C57BL/6 background were initially crossed with wild-type BALB/c mice (Vital River Laboratory). The resulting offspring heterozygous for *Cldn5*-flox and carrying the *Nphs2*-Cre transgene (*Cldn5*^flox/-^; *Nphs2*-Cre^+/−^) were backcrossed with wild-type BALB/c mice for 10 generations. In the 11th generation, progeny with genotypes *Cldn5*^flox/-^; *Nphs2*-Cre^+/−^ and *Cldn5*^flox/-^; *Nphs2*-Cre^−/−^ were identified. These were subsequently intercrossed to yield the desired *Cldn5*^flox/flox^; *Nphs2*-Cre^+/−^ knockout mice on a BALB/c background.

### DOCA/high-salt-induced hypertensive kidney injury models

Three days after right nephrectomy, 8-week-old male and female *Cldn5*-Ctrl and *Cldn5*-KO mice were anesthetized and implanted subcutaneously with a deoxycorticosterone acetate (DOCA) pellet (Innovative Research of America) (50 mg for 21 days of release) or a placebo pellet. Upon pellet implantation, all mice were provided with 0.9% normal saline as drinking water (49). Blood pressure measurements were performed weekly using a Mouse Blood Pressure System (IITC Life Science). Blood pressure was measured by averaging at least three consecutive readings taken within a 20-min period. After the experiment, kidneys were harvested and processed for histological, ultrastructural, and immunofluorescence analyses. Glomeruli were isolated for qRT-PCR and Western blot analysis.

### Enalapril administration

Six-month-old *Cldn5*-KO mice were treated with 20 mg/L enalapril maleate in the drinking water for 2 months. After treatment, the kidneys were harvested and processed for histological, ultrastructural, and immunofluorescence analyses. Glomeruli were isolated for qRT-PCR and Western blot analysis.

### Adriamycin-induced FSGS models

Eight-week-old male and female mice were randomly assigned to experimental groups using a random number table to ensure blinding. For the induction of FSGS, mice received a retroorbital injection of 10 mg/kg adriamycin (MCE), while the control group received an equivalent volume of saline (vehicle). Urine, blood, and kidney tissues were collected at 1, 2, and 4 weeks post-adriamycin injection for subsequent analyses.

### Isolation and culture of primary podocytes

Glomerulus from the *Cldn5*-ctrl and *Cldn5*-KO mice expressing a GFP-reporter were isolated using Dynabeads M-450 Tosylactivated (Invitrogen) perfusion. The isolated glomerulus was then enzymatically digested and dissociated into a single-cell suspension by using the gentleMACS Dissociators. Fluorescence-activated cell sorting (FACS) was employed to isolate GFP-positive podocytes. For Western blot and qRT-PCR analyses, podocytes were subjected to protein and RNA extraction. For podocyte culture, RPMI 1640 medium supplemented with 10% fetal calf serum was mixed in a 1:1 ratio with 3T3-L1 medium as previously described (50).

### Lentivirus infection

For gene knockdown study, shRNA hairpin oligonucleotides targeting mouse *Huwe1* (NM_021523) and *Cldn5* (NM_013805) were synthesized by Sangon Biotech. These oligonucleotides were annealed and cloned into the lentiviral vector pLKO.1 to create the *Huwe1* or *Cldn5* shRNA constructs. The targeted sequence for *Huwe1* shRNA is CCGCACTGTGTTAAACCAGAT. The targeted sequence for *Cldn5* shRNA is CAGACTACAGGCACTTTTA.

For gene overexpression study, full length sequence corresponding to coding region of *β1-integrin* (NM_010578), *Huwe1* (NM_021523), *Cldn4* (NM_009903.2), and CLDN4 chimera containing the ICL and C-term of CLDN5 were cloned into the pLVX-mcherry vector, respectively.

The lentiviral system was used to knock down *Huwe1* and *Cldn5*, and to overexpress *β1-integrin*, *Huwe1*, *Cldn4*, and the CLDN4 chimera in primary podocytes. The vector containing the target sequence or the control vector was transfected into HEK293 cells along with the packaging plasmids lenti-VSV.G, pRSV-Rev, and pMDLg/pRRE. The virus was concentrated using the protocol described previously (51). The viral pellet was then resuspended in sterile PBS and used to infect cells at a fixed titer of 10^6^ IU/ml.

### Intrarenal lentivirus delivery

The intrarenal delivery of lentivirus was performed according to a previously established protocol (52). Under anesthesia, 3-week-old male *Cldn5* knockout mice (BALB/c background) underwent a surgical procedure to introduce lentiviral particles into the left kidney. A 31-G needle was carefully inserted at the lower pole of the kidney and guided parallel to its long axis towards the upper pole. Lentiviral particles (100 μl, 1 × 10^5^ IU/μ1) were injected as the needle was slowly withdrawn. The delivered lentiviruses contained either a control vector, the wild-type CLDN5 gene, or a CLDN5-CLDN4 chimera, which was constructed by fusing the N-terminal half of CLDN5 to the C-terminal half of CLDN4. Urine and kidney tissues were harvested 3 weeks after lentiviral administration for downstream analyses.

### Expansion microscopy

The method of mouse kidney expansion microscopy was described previously (53). Briefly, 100-μm vibratome kidney slices were incubated in blocking buffer for 12 h at 4 °C. The slices were then incubated with primary antibodies against CLDN5, β1-integrin, or podocin (Table S2) for 24 h at 4 °C on a shaker. After washing, the slices were treated with secondary antibodies and DAPI. Following additional washes in PBS, the slices were incubated with freshly diluted 1 mM MA-NHS in PBS. For pre-expansion treatment, the slices were incubated in monomer solution for 1 h at 4 °C. The tissue was gently transferred to a tissue chamber and placed in a humidified environment at 37 °C for 2 h to allow gelation. After gelation, the tissue slides were digested with freshly prepared proteinase K digestion buffer and collagenase digestion buffer for 1 to 2 h at 37 °C. Following digestion, the samples were expanded in ddH_2_O, with water exchanges every 30 min for 2 h. The nuclei were restained with DAPI, and the expanded gels were mounted on poly-D-lysine–coated slides, covered with water, and imaged using Zen Software v2.3 (blue edition).

### Podocyte adhesion and spreading assays

To examine the effect of *Cldn5* deletion on podocyte adhesion on various ECM substrates, 24-well plates were coated overnight at 4 °C with Matrigel (50 mg/ml, Corning, 356,231) containing fibronectin (50 mg/ml, Corning, 354,008), collagen I (50 mg/ml, Advanced BioMatrix, 5005), collagen IV (50 mg/ml, Sigma-Aldrich, C5533), or laminin 521 (5 mg/ml, BioLamina, LN521), according to the manufacturer’s instructions. Isolated glomerulus was cultured on type I collagen-coated culture dishes for 3 days. After removing the remaining glomerular cores, podocytes were trypsinized, reseeded onto precoated 96-well plates at a density of 10^5^ cells per well in ten wells, and allowed to adhere for 2 h. After the medium and nonadherent cells were removed by tapping the inverted plate and washing with PBS for three times, the adherent cells in five of the wells were fixed with 4% paraformaldehyde and stained with 0.1% crystal violet in 2% ethanol. Absorbance was measured at 570 nm using a microplate reader (Bio-Rad Laboratories). The adherent cells in the remaining five wells were trypsinized, and the cell count was determined using a cell counter. For the spreading assay, primary podocytes were reseeded onto precoated cover slips. After a 2-h incubation at 37 °C and three washes with PBS, the adherent cells were then fixed in 2% PFA and stained with phalloidin. Cell area was quantified using NIH ImageJ software in a blinded manner, with 10 cells per animal randomly selected for analysis in each experiment.

To investigate the effect of *Cldn5* deletion on podocyte adhesion on ECM substrates with varying stiffness, gelatin methacryloyl (GelMA) hydrogels with different stiffnesses were prepared by adjusting the GelMA concentration to 5%, 10%, or 15% (w/v), corresponding to stiffness values of 0.92 kPa, 4.21 kPa, and 10.27 kPa, respectively (54, 55). GelMA powder containing the photoinitiator was purchased from EFL-Tech (China). The GelMA hydrogels were prepared by dissolving the powder in DPBS, coating 24-well plates, and then photo-crosslinking using UV light (405 nm) for 30 s. Mouse primary podocytes were reseeded onto these plates and cultured for 24 h. Cell adhesion was subsequently assessed using a CCK-8 Assay Kit (Sigma-Aldrich), a colorimetric method that measures cellular dehydrogenase activity in living cells to determine cell viability and proliferation.

### Urine and serum analysis

Mouse urine samples were collected and analyzed for urinary albumin and creatinine. Urinary albumin was measured using a mouse albumin-specific ELISA (Bethyl Laboratories), and creatinine levels were assessed using the Quantichrome Creatinine Assay Kit (Nanjing Jiancheng), following the manufacturer’s protocols. Proteinuria was expressed as μg albumin per mg creatinine.

Mouse serum samples were collected and analyzed for blood creatinine levels using the Quantichrome Creatinine Assay Kit (Nanjing Jiancheng), according to the manufacturer’s instructions.

### Histologic analysis

Mouse kidney tissues were fixed overnight at 4 °C in 4% paraformaldehyde and then embedded in paraffin. Kidney sections (5 μm thick) were prepared using standard protocols and subjected to routine histological staining, including periodic acid-Schiff (PAS) and Masson’s Trichrome Staining (MTS). Histological images were captured using a light microscope (Olympus). PAS-stained sections were analyzed for the glomerulosclerotic index (GSI) and mesangial area expansion, as described by Maric *et al*. (56). Quantification of PAS staining was performed using ImageJ v1.8.0.

For ultrastructural analysis, transmission electron microscopy (TEM) was conducted on kidney samples fixed in glutaraldehyde and embedded in epoxy resin. The sections were stained with uranyl acetate and lead citrate. TEM images were analyzed to measure the glomerular basement membrane (GBM) thickness and the number of foot processes (FP) per μm of GBM using NIH ImageJ v1.8.0 software.

### RNA extraction and qRT-PCR

Total RNA was extracted from isolated glomerulus or cultured cells using TRIzol Reagent (Invitrogen) and then reverse transcribed into cDNA using PrimeScript RT reagent Kit (Takara) for qRT-PCR. qRT-PCR was performed using SYBR Green PCR Master Mix. qRT-PCR was performed on the Applied Biosystems QuantStudio 3 Real-Time PCR System. The Table S3 contains the primer sequences used in this study. The expression levels of each mRNA were calculated after normalizing to those of β-actin. Results were expressed as 2^−ΔCt^ values with ΔCT = Ct_gene_ − Ct_β-actin_.

### Immunofluorescence staining

For immunofluorescence of kidney tissues, 7-μm frozen sections were fixed in ice-cold methanol. For immunofluorescence of primary podocytes, isolated podocytes on collagen I-coated coverslips were fixed with 4% paraformaldehyde. After blocking with 10% FBS in PBS, the samples were incubated with the appropriate primary antibodies. Secondary antibodies conjugated to FITC and/or rhodamine (TRITC) (Millipore) were used for detection. Immunofluorescence images were acquired using consistent settings for each staining combination and processed using Zen Software v2.3 (blue edition).

### Western blot analysis

Total protein extracts were obtained by lysing kidney tissues or cell samples in RIPA buffer with Complete Protease Inhibitor Cocktail Tablets (Roche Diagnostics) and phosphatase inhibitor (Sigma-Aldrich). Subcellular fractionations were isolated by using Qproteome Cell Compartment Kit from QIAGEN according to the manufacturer’s instructions. The protein was loaded and separated by SDS-PAGE, following standard procedures. The polyvinylidene fluoride membranes were blocked with 5% milk, and incubated with primary antibodies (listed in Table S2). HRP-conjugated secondary antibodies followed by ECL (Thermo Fisher) incubation allowed protein band detection. The integration of all blots images was performed on Adobe Illustrator 2021. Image J v1.8.0 was used to quantify Western blot results.

### Co-IP

Isolated glomerulus, HEK293 cells co-expressing CLDN5 and β1-integrin, α3-integrin, or β1-integrin with C-terminal deletion, as well as HEK293 cells co-expressing Flag-tagged β1-integrin and Myc-tagged CLDN were lysed in CSK buffer (50 mM Tris, pH 8.0; 150 mM NaCl; 1% Triton X-100, and protease inhibitors). Lysates were precleared by incubating with protein A/G-Sepharose (Sigma-Aldrich) before Co-IP. The precleared extracts were incubated overnight at 4 °C with antibodies against CLDN5, β1-integrin, α3-integrin, Flag, Myc, or anti-mouse IgG (as a negative control). Antibody-bound complexes were pulled down with protein A/G-Sepharose, washed three times with CSK buffer, and then boiled in 2 × SDS loading buffer at 100 °C for 10 min. Control IPs using cells transfected with either integrin or CLDN alone were performed to confirm specificity and exclude nonspecific binding. Whole-cell input lysates from single- and co-transfected cells confirmed equivalent expression of both proteins across conditions, validating transfection efficiency and interaction specificity. The samples were analyzed by SDS-PAGE and immunoblotting using the appropriate specific antibodies.

### Active β1-integrin immunoprecipitation

Proteins were extracted from isolated glomeruli using CSK buffer (50 mM Tris, pH 8.0; 150 mM NaCl; 1% Triton X-100; and protease inhibitors). The lysates were precleared using protein A/G-Sepharose and then incubated overnight at 4 °C with 2 μg of anti-active β1-integrin antibody (9EG7) or an isotype-matched IgG control. Immunocomplexes were captured using protein A/G-Sepharose, washed three times with CSK buffer, and then boiled in 2 × SDS loading buffer at 100 °C for 10 min. The samples were subsequently analyzed by SDS-PAGE and immunoblotting with a total β1-integrin antibody.

### Immunoprecipitation-mass spectrometry

Proteins were extracted from isolated *Cldn5*-ctrl and *Cldn5*-KO glomerulus using CSK buffer (50 mM Tris, pH 8.0; 150 mM NaCl; 1% Triton X-100; and protease inhibitors). Immunoprecipitation was performed using a total β1-integrin antibody. The antibody-bound protein complexes were captured and washed to remove nonspecific binders. To identify the protein interactors of β1-integrin, the immunoprecipitated samples were separated by SDS-PAGE, and a gel slice corresponding to the protein complexes was excised. The proteins in the gel were then digested with trypsin for subsequent analysis by liquid chromatography-tandem mass spectrometry (LC-MS/MS). Proteomic analysis was carried out by Shanghai Luming Biological Technology Co., Ltd (Shanghai, China). The acquired MS/MS spectra were searched against the UniProt *Mus musculus* database using the ProteomeDiscover 2.5 software for protein identification and quantification.

### β1-integrin degradation assay

Isolated podocytes were treated with cycloheximide (CHX, 20 μg/ml) for 0, 4, or 8 h, or with MG132 (10 μM) or chloroquine (CQ, 20 μM) for 8 h. After treatment, cells were harvested in RIPA lysis buffer, and protein levels were analyzed by Western blot.

### Biotinylated β1-integrin pulldown assay

To isolate biotinylated β1-integrin, cultured podocytes were incubated with NHS-SS-biotin (0.5 mg/ml, Thermo Fisher) on ice for 30 min. The biotinylation reaction was quenched by adding 50 mM Tris (pH 8.0). Following quenching, cells were washed, and then membrane proteins were extracted by incubating the cell pellet in CSK buffer (150 mM NaCl; 1% Triton X-100; 50 mM Tris, pH 8.0; and a protease inhibitor cocktail) at 4 °C with gentle rotation for 1 h. The resulting membrane extract was clarified by centrifugation at 5000×*g* for 10 min at 4 °C. The membrane extract was then incubated with NeutrAvidin Agarose to capture biotinylated proteins. Following incubation, the NeutrAvidin-bound biotinylated proteins were eluted using SDS sample buffer containing β-mercaptoethanol. The eluted samples were subjected to SDS-PAGE under denaturing conditions, followed by Western blot analysis to assess the expression levels of β1-integrin.

### Cell surface β1-integrin internalization assay

Pulse-chase biotinylation experiments were performed as described previously (57). In briefly, podocyte cell surface proteins were biotinylated ice using NHS-SS-biotin (0.5 mg/ml, Thermo Fisher) on ice for 30 min. The internalization of cell surface proteins was induced by placing the cells at 37 °C for 2 h. After the internalization period, cells were returned to 4 °C and treated twice with 20 mM 2-mercaptoethane sulfonate sodium for 5 min each to strip biotin from non-internalized proteins. This was followed by a 15-min treatment with 20 mM iodoacetamide to quench any remaining 2-mercaptoethane sulfonate sodium. Cells were then lysed in RIPA buffer and incubated on ice for 30 min, followed by centrifugation at 14,000 rpm for 10 min at 4 °C to obtain soluble lysates. Biotinylated proteins were purified using monomeric avidin agarose according to the manufacturer’s instructions, and proteins were eluted in reducing SDS sample buffer and subjected to Western blot analysis. Stripping controls were included by keeping a separate group of cells at 4 °C after biotin labeling before stripping and lysing.

### Adhesion complexes isolation

Adhesion complexes were isolated following a modified version of a previously described protocol (58). Briefly, isolated podocytes were seeded onto type I collagen-coated culture dishes and allowed to spread for 2 h. Cells were then incubated for 3 min with 6 mM dimethyl-3,3′-dithiobispropionimidate (DTSSP; Sigma-Aldrich) dissolved in Advanced DMEM (Sigma-Aldrich) to crosslink proteins. The crosslinking reaction was quenched by the addition of Tris-HCl (pH 8.5). After additional washes in PBS, cell bodies were removed by lysis in RIPA buffer (25 mM Tris-HCl, 150 mM NaCl, 1% Triton X-100, 0.2% SDS, 0.5% sodium deoxycholate, pH 7.5) containing protease inhibitors for 3 min at 4 °C, followed by application of hydrodynamic force using a waterpik (2 × 10 s washes with PBS). Focal adhesion complexes remaining bound to the substrate were collected in reducing sample buffer (50 mM Tris-HCl, pH 6.8, 10% glycerol, 4% SDS, 0.004% bromophenol blue, 8% β-mercaptoethanol) and incubated at 70 °C for 10 min. Western blotting was performed subsequently.

### FRET assay

HEK293 cells were co-transfected with constructs encoding either CLDN5-CFP or CLDN4-CFP along with β1-integrin-YFP. For fluorescence imaging, CFP was excited using a 405-nm laser, and its emission was recorded in the range of 460 to 492 nm. YFP was excited with a 514-nm laser, and its emission was monitored from 526 to 589 nm. To assess fluorescence recovery after photobleaching, YFP was photobleached with the 514-nm laser at 90% power for 1 min. Following photobleaching, images of CFP and YFP fluorescence were captured using the appropriate filter sets. Data were collected from 10 distinct cells across various fields of view on the same coverslip. The mean CFP fluorescence intensity before and after photobleaching was quantified using ZEN software.

### Cell volume measurements

Isolated podocytes were prepared for cell volume assessment. To visualize cellular boundaries, the plasma membrane was stained with Alexa Fluor 488-conjugated wheat germ agglutinin (Thermo Fisher Scientific). Cell nuclei were counterstained with DAPI. Confocal microscopy images were acquired using a Zeiss confocal microscope. Z-stacks, comprising a series of optical slices through the cells from top to bottom, were captured. These 2D optical slices were subsequently reconstructed into a 3D representation. Cell volume was then quantitatively estimated using the ZEN software (Zeiss). In parallel, the approximate size of isolated podocytes was assessed using forward scatter (FSC) measurements on a BD flow cytometer. Flow cytometry data were analyzed and prepared for presentation using FlowJo software (version 8.1).

### Bimolecular fluorescence complementation (BiFC) assay

As described previously (59), CLDN4, CLDN5, CLDN8, CLDN14, CLDN16, CLDN19, or a CLDN chimera (listed in Table S4) cloned into the pBiFC-VN155 (I152 L) plasmid, along with α3-integrin or β1-integrin cloned into the pBiFC-VC155 plasmid, were co-transfected into HEK293 cells using Lipofectamine 3000. Forty-eight hours post-transfection, cells were fixed with 4% paraformaldehyde and stained with DAPI. Cell images were captured using the same exposure settings and processed with Zen Software v2.3 (Blue Edition).

### Statistical analysis

The significance of differences between groups was tested by GraphPad Prism 8.0 Software. The normality of data distribution was assessed using the Kolmogorov-Smirnov test. Two-group comparisons were performed using the two-tailed Student’s *t* test for normally distributed data and the Mann-Whitney test for non-normally distributed data. Differences among multiple groups with a single variable were analyzed using one-way ANOVA followed by *post hoc* Tukey’s test. For comparing multiple groups with more than one variable, two-way ANOVA with *post hoc* Tukey’s test was employed. All Data are presented as mean ± SD. *p*-values < 0.05 were interpreted as statistically significant. Other details, such as the number of replicates and the level of significance are mentioned in figure legends.

## Data availability

The data supporting the findings of this study are available in the main manuscript or the supporting information. In addition, we will share models, protocols, methods, and other useful materials and resources related to the article as far as possible. There are no large data files to be shared via online resources.

## Supporting information

This article contains supporting information.

## Conflict of interest

The authors declare that they do not have any conflicts of interest with the content of this article.
