# Supplementary material for: CLDN5 as a novel modulator of podocyte adhesion to extracellular matrix via β1-integrin binding
Source: J Biol Chem. 2026 Jan 13;302(3):111163. doi: 10.1016/j.jbc.2026.111163 (PMC12887804; doi:10.1016/j.jbc.2026.111163)
Supplement: Supplementary Material [file mmc1.pdf]

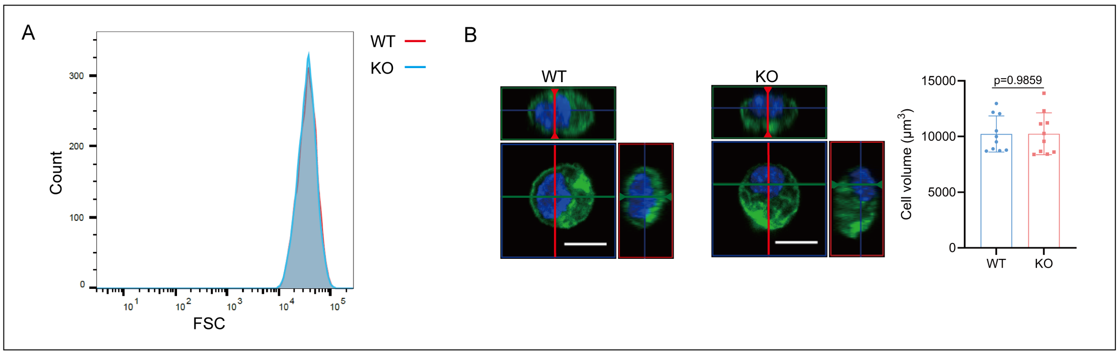

Figure S1. Quantitative assessment of cell volume in WT and *Cldn5*-KO podocytes. (A) Cell volume distribution analysis by flow cytometry. Forward scatter (FSC) is used as a surrogate for cell size. (B) Representative 3D confocal reconstructions illustrating cell volume of isolated podocytes. Nuclei are counterstained with DAPI (blue). n = 10 cells analyzed per group. Scale bars, 10 μm. Data are presented as mean ± SD. Statistical significance was determined by two-tailed Student's unpaired t test analysis.

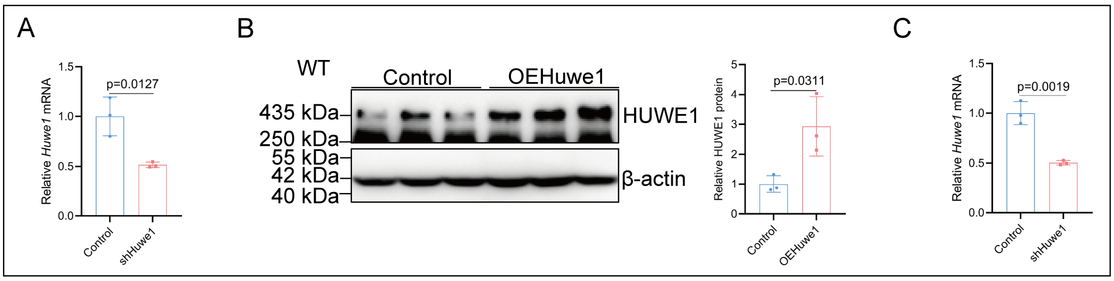

Figure S2. CLDN5 protects β1-integrin from HUWE1-mediated ubiquitination and degradation. (A) qRT-PCR analysis of HUWE1 mRNA in the isolated WT podocytes treated with lentivirus carrying either control- or *Huwe1*-shRNA (n = 3). (B) Western blot with densitometric analysis of HUWE1 in the isolated WT podocytes treated with lentivirus carrying either control or *Huwe1* coding sequence (n = 3). (C) qRT-PCR analysis of HUWE1 mRNA in the isolated *Cldn5*-KO podocytes treated with lentivirus carrying either control- or *Huwe1*-shRNA (n = 3). Data are presented as mean ± SD. Statistical significance was determined by two-tailed Student's unpaired t test analysis.

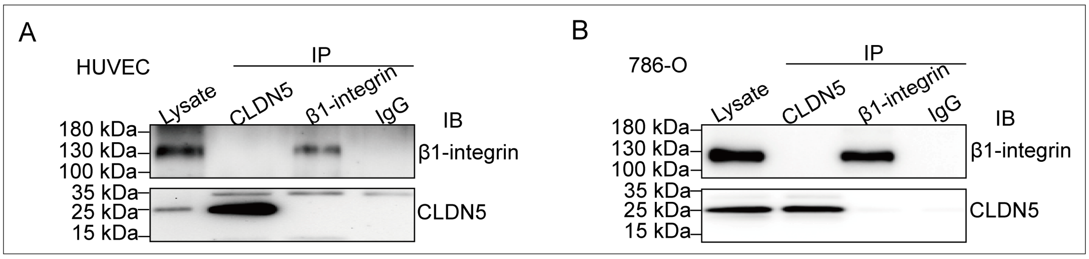

Figure S3. Co-immunoprecipitation (Co-IP) analysis of CLDN5 and β1-integrin interaction in non-podocyte cell lines. (A) Co-IP in human umbilical vein endothelial cells (HUVEC). (B) Co-IP in human renal clear cell adenocarcinoma cells (786-O). For each Co-IP analysis, the antibody used for immunoprecipitation is indicated above the lanes, and the antibody used for Western blot is shown to the right of the blots.

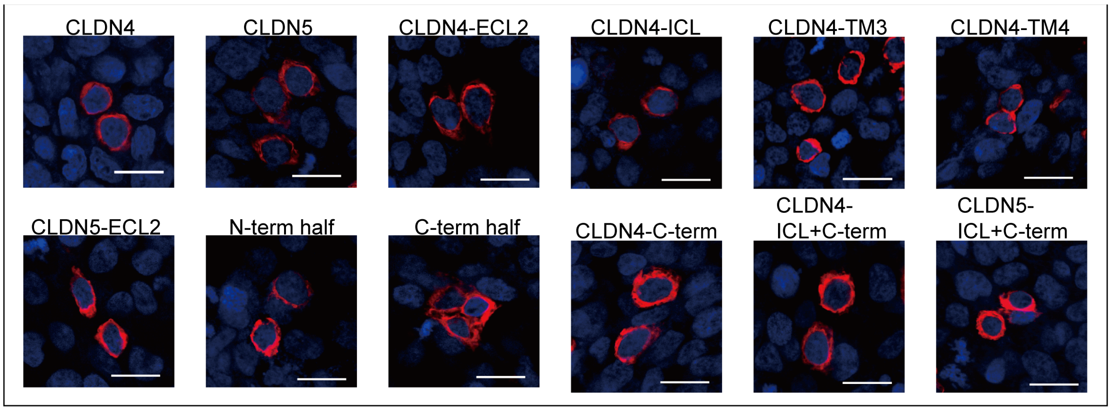

Figure S4. Fluorescence images of CLDN4, CLDN5, and CLDN chimeras in HEK293 cells transfected with plasmids expressing CLDN4, CLDN5, or CLDN chimeras fused with mCherry. Scale bar: 20 μm.

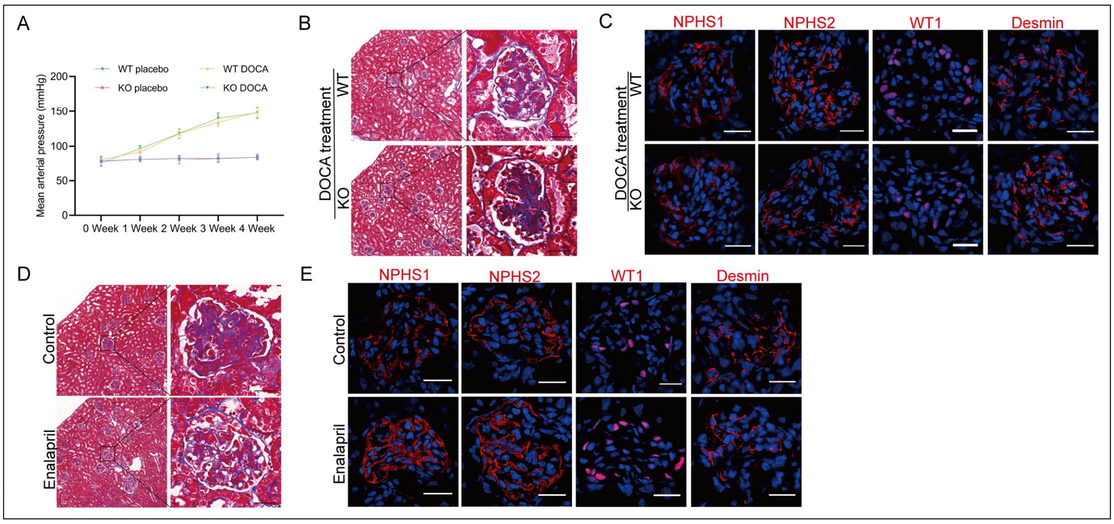

Figure S5. CLDN5 deficiency in podocytes exacerbates hypertensive nephropathy. (A) Mean arterial pressure change of WT and *Cldn5*-KO mice subjected to DOCA/high-salt-induced hypertension (n = 6). (B) Masson's trichrome staining (MTS) in kidneys from WT and *Cldn5*-KO mice subjected to DOCA/high-salt-induced hypertension. Scale bars, 20 μm. (C) Immunofluorescence of NPHS1, NPHS2, WT1, and Desmin in kidneys from WT and *Cldn5*-KO mice subjected to DOCA/high-salt-induced hypertension. Scale bars, 20 μm. (D) MTS in kidneys from 8-month-old *Cldn5*-KO mice with or without enalapril treatment. Scale bars, 20 μm. (E) Immunofluorescence of NPHS1, NPHS2, WT1, and Desmin in kidney sections from 8-month-old *Cldn5*-KO mice with or without enalapril treatment. Scale bars, 20 μm. Data are presented as mean ± SD.

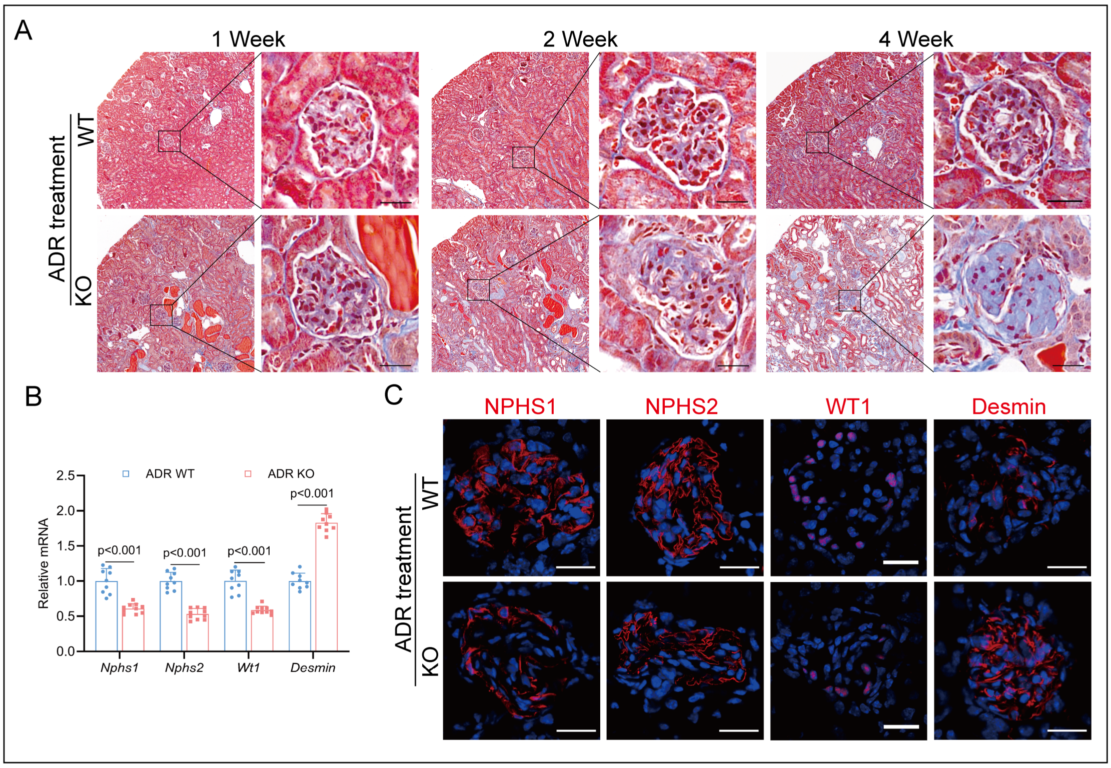

Figure S6. Podocyte CLDN5 deficiency renders male C57BL/6 mice susceptible to adriamycin-induced nephropathy. (A) MTS in kidneys from WT or *Cldn5*-KO C57BL/6 mice following adriamycin treatment. Scale bars, 20 μm. (B) qRT-PCR analysis of *Nphs1*, *Nphs2*, *Wt1*, and *Desmin* in glomerulus from WT or *Cldn5*-KO C57BL/6 mice following adriamycin treatment (n = 9). (C) Immunofluorescence of NPHS1, NPHS2, WT1, and Desmin in kidney sections from the WT or *Cldn5*-KO C57BL/6 mice following adriamycin treatment. Scale bars, 20 μm. Data are presented as mean ± SD. Statistical significance was determined by two-way ANOVA followed by Tukey's post-test.

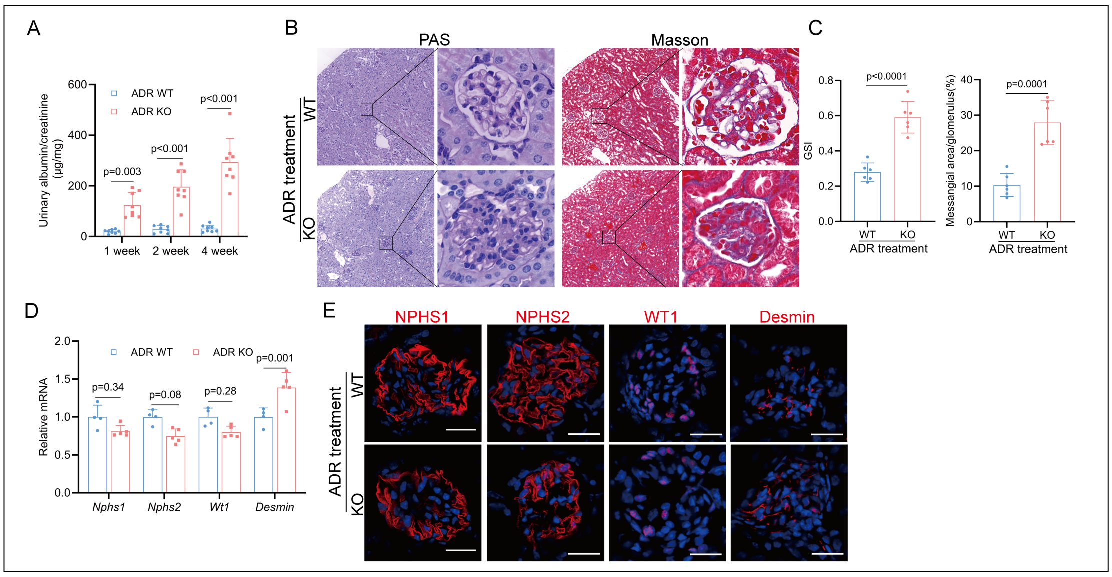

Figure S7. Deletion of CLDN5 in podocytes sensitizes female C57BL/6 mice to adriamycin-induced nephropathy. (A) Albumin-to-creatinine ratio (µg/mg) in urine from female WT or *Cldn5*-KO mice following adriamycin treatment (n = 8). (B and C) PAS staining and MTS (B), glomerular sclerosis index (GSI) and mesangial area expansion (C) in kidneys from female WT or *Cldn5*-KO mice following adriamycin treatment (n = 6 biologically independent animals, 10 glomeruli per mouse were analyzed). Scale bars, 20 µm. (D) qRT-PCR analysis of *Nphs1*, *Nphs2*, *Wt1*, and *Desmin* in glomerulus from female WT or *Cldn5*-KO mice following adriamycin treatment (n = 4). (E) Immunofluorescence of NPHS1, NPHS2, WT1, and Desmin in kidney sections from the female WT or *Cldn5*-KO mice following adriamycin treatment. Scale bars, 20 µm. Data are presented as mean ± SD. Statistical significance was determined by two-way ANOVA followed by Tukey's post-test (A and D), two-tailed Student's unpaired t test (C).

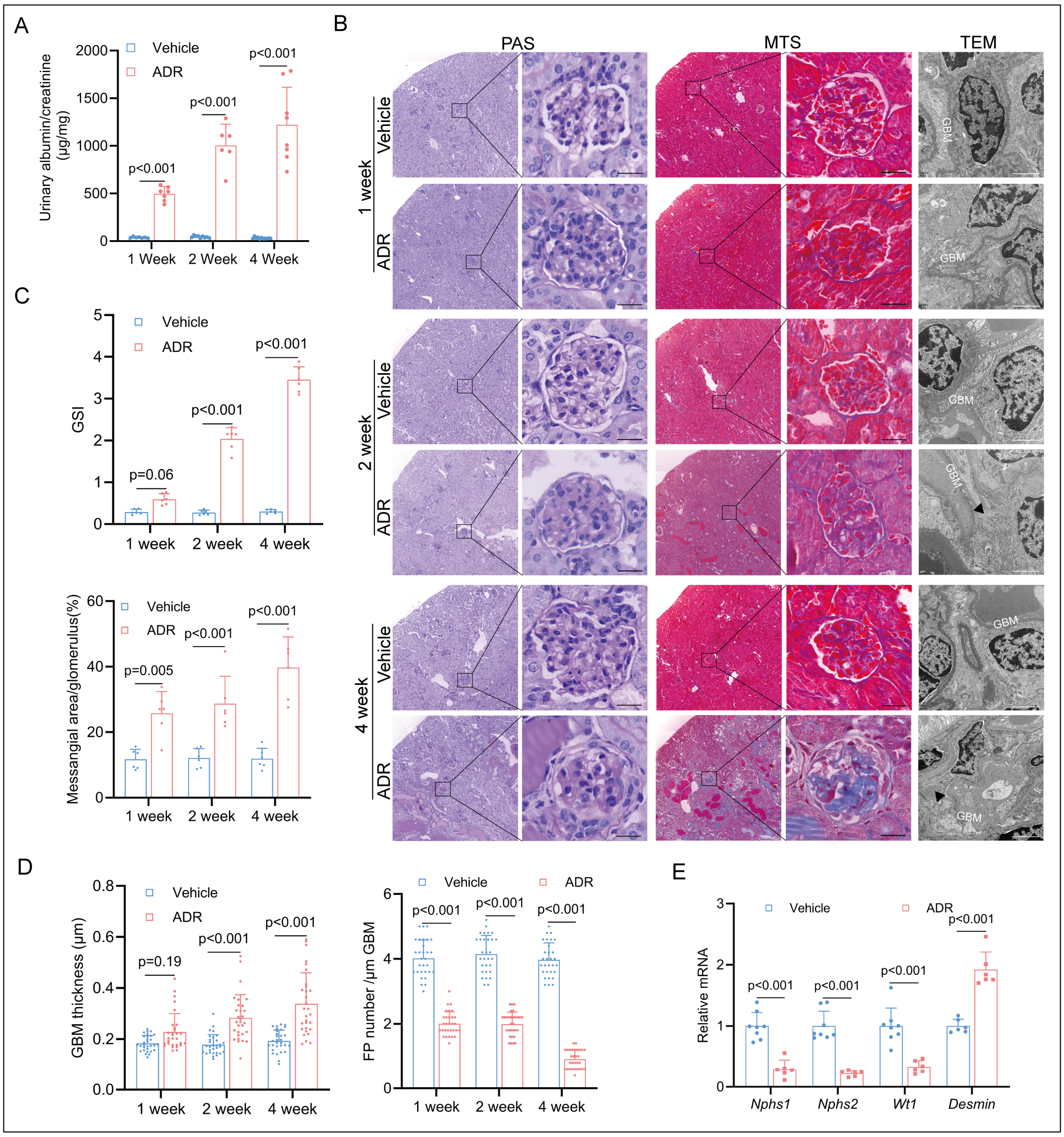

Figure S8. Validation of the adriamycin-induced nephropathy model in BALB/c mice. (A) Albumin-to-creatinine ratio (μg/mg) in urine from BALB/c mice following adriamycin treatment (n = 6). (B-D) Morphological examinations of glomerular changes by PAS staining (n = 6 biologically independent animals, 10 glomeruli per mouse were analyzed), MTS, and TEM analyses (n = 3 biologically independent animals, 10 images per group) in kidneys from BALB/c mice following adriamycin treatment. Black triangles highlight foot process effacement. Scale bars: 20 μm for PAS staining images and 2 μm for TEM images. (E) qRT-PCR analysis of *Nphs1*, *Nphs2*, *Wt1*, and *Desmin* in glomerulus from BALB/c mice following

adriamycin treatment (n = 6). Data are presented as mean ± SD. Statistical significance was determined by two-way ANOVA followed by Tukey's post-test (A, C-E).

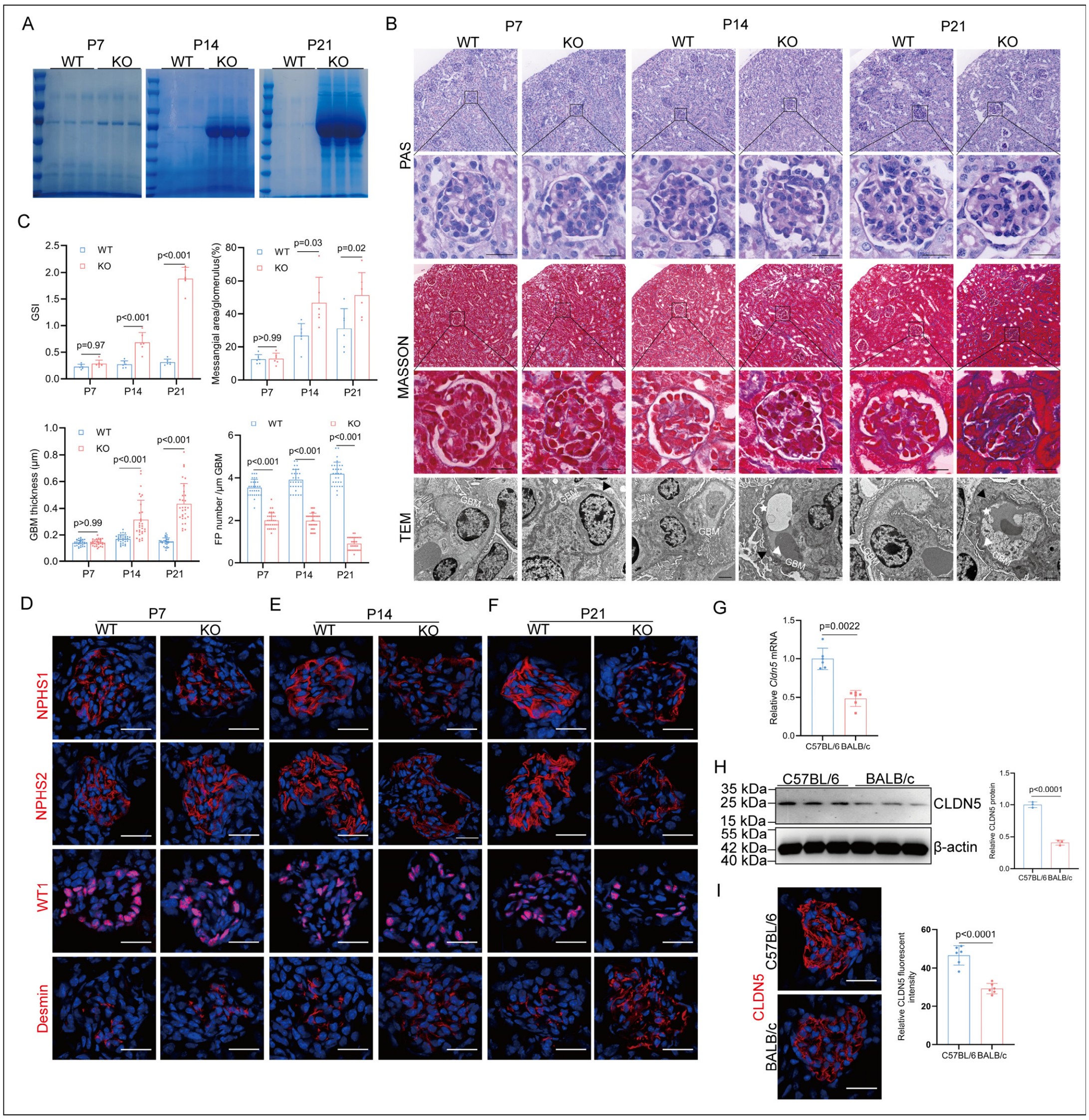

Figure S9. *Cldn5* knockout triggers early proteinuria and progressive glomerulopathy in susceptible BALB/c mice. (A) Coomassie-stained gel displaying albumin levels in urine from 7-, 14-, and 21-day-old WT and *Cldn5*-KO BALB/c mice (2 μl urine per lane, n = 3). (B and C) Morphological examinations of glomerular changes by PAS staining (n = 6 biologically independent animals, 10 glomeruli per mouse were analyzed), MTS, and TEM analyses (n = 3 biologically independent animals, 10 images per group) in kidneys from 7-, 14-, and 21-day-old WT and *Cldn5*-KO BALB/c mice. White stars indicate GBM spikes, white triangles denote GBM splitting, and black triangles highlight foot process effacement. Scale bars: 20 μm for PAS staining images and 2 μm for TEM images. (D-F) Immunofluorescence of NPHS1, NPHS2,

WT1, and Desmin in kidney sections from WT or *Cldn5*-KO BALB/c mice at 7 (D), 14 (E), and 21(F) days of age. Scale bars, 20 µm. (G-I) qRT-PCR analysis (G, n = 6), Western blot with densitometric analysis (H, n = 3), and immunofluorescence (I) of CLDN5 in the glomerulus of 4-week-old C57BL/6 and BALB/c mice. Scale bars, 20 µm. Data are presented as mean ± SD. Statistical significance was determined by two-way ANOVA followed by Tukey's post-test (C), nonparametric Mann-Whitney test (G), two-tailed Student's unpaired t test (H and I).

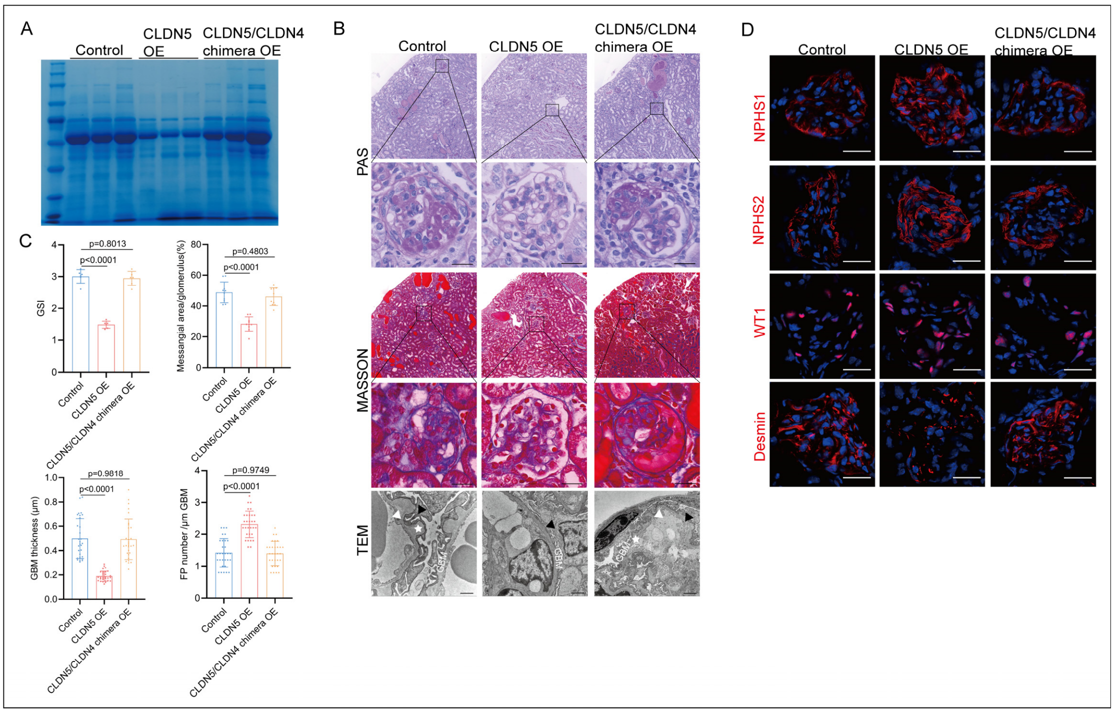

Figure S10. CLDN5 restoration alleviates renal injury in *Cldn5*-KO mice. (A) Coomassie-stained gel displaying albumin levels in urine from *Cldn5*-KO BALB/c mice treated with AAV carrying either control, wild-type CLDN5, or CLDN5–CLDN4 chimera (2 μl urine per lane, n = 3). (B and C) Morphological examinations of glomerular changes by PAS staining (n = 6 biologically independent animals, 10 glomeruli per mouse were analyzed), MTS, and TEM analyses (n = 3 biologically independent animals, 10 images per group) in kidneys from *Cldn5*-KO BALB/c mice treated with AAV carrying either control, wild-type CLDN5 or CLDN5–CLDN4 chimera. White stars indicate GBM spikes, white triangles denote GBM splitting, and black triangles highlight foot process effacement. Scale bars: 20 μm for PAS staining images and 2 μm for TEM images. (D) Immunofluorescence of NPHS1, NPHS2, WT1, and Desmin in kidney sections from *Cldn5*-KO BALB/c mice treated with AAV carrying either control, wild-type CLDN5 or CLDN5–CLDN4 chimera. Scale bars, 20 μm. Data are presented as mean ± SD. Statistical significance was determined by one-way ANOVA followed by Tukey's post-test.

Table S1: Reagents list

**Product name**	**Cat. NO.**	**Company**
DMEM	C3103-0500	vivacell biosciences
RPMI 1640	C3001-0500	vivacell biosciences
FBS	10099141C	Gibco
Penicillin/Streptomycin Solution	MA0110	Meilunbio
Puromycin	HY-B1743A	MCE
Q5® Site-Directed Mutagenesis Kit	E0554S	NEB
Trypsin-EDTA Solution	E607002	Sangon Biotech
Lipofectamine 3000	L3000015	lnvitrogen
TRIzol	15596018	Invitrogen
MG132	HY-13259	MCE
Chloroquine (CQ)	HY-17589A	MCE
Cycloheximide (CHX)	HY-12320	MCE
Cell Counting Kit-8 Assay	96992	Sigma-Aldrich
PrimeScript™ RT reagent Kit with gDNA Eraser	RR047A	Takara
SYBR Green PCR Master Mix	K0251	Thermo Scientific
O.C.T. Compound	4583	SAKURA
BSA	V900933	Sigma-Aldrich
Tween 20	P9416	Sigma-Aldrich
DAPI	10236276001	Sigma-Aldrich
Triton X-100	T8787	Sigma-Aldrich
RIPA lysis buffer	C500007	Sangon Biotech
cOmplete Protease Inhibitor Cocktail tablets	11697498001	Roche Diagnostics
phosphatase inhibitor	P0044-1ML	Sigma-Aldrich
TaKaRa BCA Protein Assay Kit	T9300A	Takara
SuperSignal™ West Pico PLUS (ECL)	34580	Thermo Fisher
Protein A/G-sepharose	80106G	Invitrogen
Deoxycorticosterone acetate (DOCA)	M-121-50mg/25pellets	Innovative Research of America
Enalapril maleate	HY-B0331A	MCE
Dynabeads™ M-450 Tosylactivated	14013	Invitrogen
Multi Tissue Dissociation Kits	130-110-204	Miltenyi
TEMPO	426369-1G	Sigma-Aldrich
Methacrylic Acid-NHS	730300-1G	Sigma-Aldrich
Ammonium Persulfate (APS)	248614-5G	Sigma-Aldrich
Tetramethylethylenediamine (TEMED)	T22500-5ML	Sigma-Aldrich
40% Acrylamide	A4058-100ML	Sigma-Aldrich
Bisacrylamide	M7279-25G	Sigma-Aldrich
Sodium Acrylate	sc-236893B	Santa Cruz
8 M Guanidine-HCl	G7294-100ML	Sigma-Aldrich
Collagenase	C7926-100MG	Sigma-Aldrich

Proteinase K	HY-108717	MCE
Matrigel	356231	Corning
Fibronectin	354008	Corning
Collagen I	5005	Advanced BioMatrix
Collagen IV	C5533	Sigma-Aldrich
Laminin 521	LN521	BioLamina
Gelatin methacryloyl (GelMA)	EFL-GM-30	EFL-Tech
Mouse Albumin ELISA Kit	E99-134	Bethyl Laboratories
Quantichrome Creatinine Assay Kit	C011-2-1	Nanjing Jiancheng
Qproteome Cell Compartment Kit	37502	QIAGEN
NHS-SS-biotin	21441	Thermo Fisher
2-mercaptoethane sulfonate sodium	M1511-5G	Sigma-Aldrich
Iodoacetamide	I6125-5G	Sigma-Aldrich
Monomeric avidin agarose	20228	Pierce
Alexa Fluor™ 488 Phalloidin	A12379	Invitrogen
Dimethyl-3,3′-dithiobispropionimidate	D2388	Sigma-Aldrich
Advanced DMEM	12491015	Gibco
Tris-HCl (pH 8.5)	ST785-100ml	Beyotime
Leupeptin	L2884	Sigma-Aldrich
Aprotinin	A1153	Sigma-Aldrich
AEBSF	A8456	Sigma-Aldrich
Glycerol	G5516	Sigma-Aldrich
SDS	L3771	Sigma-Aldrich
Bromophenol blue	114391	Sigma-Aldrich
β-mercaptoethanol	444203	Sigma-Aldrich

Table S2: primary antibody list

Target	Product name	Host	Applications	Cat. NO.	Company
CLDN5	Claudin 5 Polyclonal Antibody	Rabbit	CoIP	34-1600	Invitrogen
CLDN5	Claudin 5 Monoclonal Antibody	Mouse	WB, IF	35-2500	Invitrogen
β1-integrin	Anti-Integrin beta 1 Antibody	Rabbit	WB, IF, CoIP	ab183666	Abcam
β1-integrin	CD29 (Integrin beta 1) Monoclonal Antibody	Armenian hamster	IF	14-0291-82	Invitrogen
Active β1-integrin	Rat Anti-Mouse CD29	Rat	IP	553715	BD
HUWE1	HUWE1 Recombinant Rabbit Monoclonal Antibody	Rabbit	WB, IF	MA5-44447	Invitrogen
HUWE1	Anti-HUWE1/Mule antibody	Rabbit	WB, CoIP	ab70161	Abcam
Laminin α5	Anti-Laminin alpha 5/LAMA5 Antibody	Rabbit	WB	ab184330	Abcam
NPSH1	Mouse Nephrin Antibody	Goat	WB, IF	AF3159	R&D
NPSH2	Anti-NPHS2 Antibody	Rabbit	WB, IF	ab50339	Abcam
WT1	Anti-Wilms Tumor Protein Antibody	Rabbit	WB, IF	ab89901	Abcam
Desmin	Anti-Desmin Antibody	Rabbit	WB, IF	ab15200	Abcam
Talin-1	Anti-Talin 1 Antibody	Rabbit	WB	ab71333	Abcam
Vinculin	Monoclonal Anti-Vinculin Antibody	Mouse	WB	V4505	Sigma
ITGA3	Integrin α3/ITGA3/CD49c Antibody	Mouse	WB, CoIP	sc-374242	Santa cruz Biotechnology
Ubiquitin	Ubiquitin Recombinant	Rabbit	WB	701339	Invitrogen

	Rabbit Monoclonal Antibody				
Myc-tag	MYC tag Monoclonal antibody	Mouse	WB, CoIP	60003-2-Ig	Proteintech
Flag-tag	DYKDDDDK tag Monoclonal antibody	Mouse	WB, CoIP	66008-4-Ig	Proteintech
Myc-tag	MYC tag Polyclonal antibody	Rabbit	WB, CoIP	16286-1-AP	Proteintech
β-actin	Anti-beta Actin Antibody	Mouse	WB	ab8226	Abcam
GAPDH	Anti-GAPDH Antibody	Rabbit	WB	ab181602	Abcam

Table S3: Primer sequences used in this study

Gene	Forward	Reverse
Cldn5	GTTAAGGCACGGGTAGCACT	GTACTTCTGTGACACCGGCA
Nphs1	CAGCTGCTAGTCTGCGAGG	ATCAATGACAGGAGGTCCTG
Nphs2	GTGAGGAGGGCACGGAAGT	TAATCCAGAGGGCTTGATGC
Wt1	TTCACCTTGCACTTCTCGGG	TGACCGTGCTGTATCCTTGG
Desmin	GATAGACGACCTGCAGAGGC	CATACTGAGCCCGGATGTCC
β1-integrin	GCAAATGCCAAATCTTGCGG	AATGAGCCAAAGCCAATGCG
Huwe1	AGTTGCTTGAGAGGGATGGC	TGGAATCTTCTCGGTTGGCC
Cldn4	GCCTCTGGATGAACTGCGT	CACTTGCCCCCTACCACTG
Cldn8	GTCTTGGCTTTCTTGGCTTTCA	AGAAGATGATTCCGGCTGTCAG
Cldn14	GGCATGGTAGGAACGCTCAT	TGCCACACACATTCCATCCA
Cldn16	ATTCATCACCCTGCTCCTTGG	TCGACGTAAACATCCACAGCA
Cldn19	CTCTACGAAGGGCTGTGGATG	GTGCTGACTGGATATGACCGT
β-actin	GGCTGTATTCCCCTCCATCG	CCAGTTGGTAACAATGCCATGT
β1-integrin-K752R	GCTGATTTGGcgcCTTTTAATGATAATTCATGACAGAAG	AGCAAGGCAAGGCCA
β1-integrin-K765R	GGAATTTGCTcgcTTTGAAAAGGAGAAAATG	CTTCTGTCATGAATTATCATTAAAAG
β1-integrin-K768R	TAAATTTGAAcgcGAGAAAATGAATGCCAAGTGG	GCAAATTCCCTTCTGTC
β1-integrin-K770R	TGAAAAGGAGcgcATGAATGCCAAG	AATTTAGCAAATTCCCTTCTG
β1-integrin-K774R	AATGAATGCCcgcTGGGACACGGG	TTCTCCTTTTCAAATTTAGC
β1-integrin-K784R	TCCTATTTACcgcAGTGCCGTGAC	TTTTCACCCGTGTCC
β1-integrin-K794R	GGTCAATCCGcgcTATGAGGGAAAAGAATTCTG	ACAGTTGTCACGGCAC
β1-integrin-K798R	GTATGAGGGAcgcGAATTCTGCAGTCGAC	TTCGGATTGACCACAG
β1-integrin-C	GAATTCTGCAGTCGAC	ATAAATAGGATTTTCACCCG

Table S4: Amino acid sequence of CLDN chimeras used in this study

CLDN chimera	CLDN4 sequence	CLDN5 sequence
CLDN4-ECL2	…TAHNIIQDFYNPLVASGQKREM …	MGSAALEILGLVLCLVGWGGLIL ACGLPMWQVTAFLDHNIVTAQT TWKGLWMSCVVQSTGHMQCKV YDSVLALSTEVQAARALTVSAVL LAFVALFVTLAGAQCTTCVAPGP AKARVALTGGVLYLFCGLLALVP LCWFANIV…LGAALYIGWAATA LLMVGGCLLCCGAWVCTGRPDL SFPVKYSAPRRPTATGDYDKKNY V
CLDN5-ECL2	MASMGLQVMGIALAVLGWLAVM LCCALPMWRVTAFIGSNIVTSQTI WEGLWMNCVVQSTGQMQCKVY DSLLALPQDLQAARALVIISIIVAAL GVLLSVVGGKCTNCLEDESAKAK TMIVAGVVFLLAGLMVIVPVSW… GASLYVGWAASGLLLLGGGLLCC NCPPRTDKPYSAKYSAARSAAASN YV	…VREFYDPSVPVSQKYE…
N-term half	MASMGLQVMGIALAVLGWLAVM LCCALPMWRVTAFIGSNIVTSQTI WEGLWMNCVVQSTGQMQCKVY DSLLALPQDLQAARALVIISIIVAAL GVLLSVVGG…	…VREFYDPSVPVSQKYELGAAL YIGWAATALLMVGGCLLCCGAW VCTGRPDLSFPVKYSAPRRPTAT GDYDKKNYV
C-term half	…KCTNCLEDESAKAKTMIVAGVV FLLAGLMVIVPVSWTAHNIIQDFY NPLVASGQKREMGASLYVGWAAS GLLLLGGGLLCCNCPPRTDKPYSA	MGSAALEILGLVLCLVGWGGLIL ACGLPMWQVTAFLDHNIVTAQT TWKGLWMSCVVQSTGHMQCKV YDSVLALSTEVQAARALTVSAVL

	KYSAARSAAASNYV	LAFVALFVTLAGAQCTTCVAPGP<br>AKARVALTGGVLYLFCGLLALVP<br>LCWFANIV…
CLDN4-ICL	…KCTNCLEDESAKAKT…	MGSAALEILGLVLCLVGWGGLIL<br>ACGLPMWQVTAFLDHNIVTAQT<br>TWKGLWMSCVVQSTGHMQCKV<br>YDSVLALSTEVQAARALTVSAVL<br>LAFVALFVTLAGA…VLYLFCGLL<br>ALVPLCWFANIVVREFYDPSVPV<br>SQKYELGAALYIGWAATALLMV<br>GGCLLCCGAWVCTGRPDLSFPV<br>KYSAPRRPTATGDYDKKNYV
CLDN4-TM3	…MIVAGVVFLLAGLMVIVPVSW<br>…	MGSAALEILGLVLCLVGWGGLIL<br>ACGLPMWQVTAFLDHNIVTAQT<br>TWKGLWMSCVVQSTGHMQCKV<br>YDSVLALSTEVQAARALTVSAVL<br>LAFVALFVTLAGAQCTTCVAPGP<br>AKARVALTGG…VREFYDPSVPV<br>SQKYELGAALYIGWAATALLMV<br>GGCLLCCGAWVCTGRPDLSFPV<br>KYSAPRRPTATGDYDKKNYV
CLDN4-TM4	…GASLYVGWAASGLLLLGGGLL<br>…	MGSAALEILGLVLCLVGWGGLIL<br>ACGLPMWQVTAFLDHNIVTAQT<br>TWKGLWMSCVVQSTGHMQCKV<br>YDSVLALSTEVQAARALTVSAVL<br>LAFVALFVTLAGAQCTTCVAPGP<br>AKARVALTGGVLYLFCGLLALVP<br>LCWFANIVVREFYDPSVPVSQKY<br>E…LCCGAWVCTGRPDLSFPVKY

		SAPRRPTATGDYDKKNYV
CLDN4-C-term	…CCNCPPRTDKPYSAKYSAARSA AASNYV…	MGSAALEILGLVLCLVGWGGLIL ACGLPMWQVTAFLDHNIVTAQT TWKGLWMSCVVQSTGHMQCKV YDSVLALSTEVQAARALTVSAVL LAFVALFVTLAGAQCTTCVAPGP AKARVALTGGVLYLFCGLLALVP LCWFANIVVREFYDPSVPVSQKY ELGAALYIGWAATALLMVGGCL …
CLDN4-ICL/C-term	…KCTNCLEDESAKAKT…CCNCPP RTDKPYSAKYSAARSAAASNYV	MGSAALEILGLVLCLVGWGGLIL ACGLPMWQVTAFLDHNIVTAQT TWKGLWMSCVVQSTGHMQCKV YDSVLALSTEVQAARALTVSAVL LAFVALFVTLAGAQCTTCVAPGP AKARVALTGGVLYLFCGLLALVP LCWFANIV…LGAALYIGWAATA LLMVGGCL…
CLDN5-ICL/C-term	…VREFYDPSVPVSQKYE…LCCGA WVCTGRPDLSFPVKYSAPRRPTAT GDYDKKNYV	MASMGLQVMGIALAVLGWLAV MLCCALPMWRVTAFIGSNIVTSQ TIWEGLWMNCVVQSTGQMQCK VYDSLLALPQDLQAARALVIISII VAALGVLLSVVGG…MIVAGVVF LLAGLMVIVPVSWTAHNIIQDFY NPLVASGQKREMGASLYVGWAA SGLLLLGGGLL…
